# Ejecta‐Modulated Bubble Dynamics Play a Dominant Role in Stone Retropulsion

**DOI:** 10.1002/advs.202516280

**Published:** 2026-02-20

**Authors:** Obed S. Isaac, Arpit Mishra, Georgy N. Sankin, Junqin Chen, Pei Zhong

**Affiliations:** ^1^ Thomas Lord Department of Mechanical Engineering and Materials Science Duke University Durham USA; ^2^ Department of Biomedical Engineering Tulane University New Orleans USA

**Keywords:** bubble dynamics, cavitation, ejecta, laser‐lithotripsy, retropulsion

## Abstract

Cavitation bubbles generated by laser absorption in liquids collapse violently, producing high‐speed jets, toroidal bubbles, and shock waves that induce material erosion and object displacement. In laser lithotripsy, this phenomenon causes kidney stone migration (retropulsion) which reduces procedural efficiency and requires frequent repositioning of the fiber tip. Retropulsion has traditionally been attributed to recoil momentum from laser‐generated ejecta; however, our experiments demonstrate that vapor bubble dynamics, rather than ejecta recoil, predominantly govern stone motion, while ejecta modulate bubble morphology and collapse asymmetry. A clinical Ho:YAG laser system was used to deliver pulses at varying stand‐off distances to freely suspended Begostone phantoms. Ultra‐high‐speed imaging up to 5 million frames per second, complemented by optical coherence tomography, captured the coupled evolution of bubble lifecycles, ejecta behavior, crater formation, and stone displacement in both air and water. Analysis reveals that while ejecta content decreases with continued ablation, bubble‐driven forces persist and dominate retropulsion. Asymmetric crater geometries formed by earlier pulses influence subsequent bubble shape and collapse, altering both the magnitude and direction of stone movement. A dimensionally consistent empirical model based on geometric, temporal, and morphological parameters quantitatively describes these effects. The results demonstrate that ejecta‐modulated bubble dynamics‐wherein ejecta shape the crater geometry and induce bubble asymmetry that guides collapse‐jet formation and the resulting bulk fluid momentum transfer‐constitute the primary physical mechanism underlying stone retropulsion, providing new insights for optimizing laser delivery strategies in lithotripsy and for controlled cavitation applications in medicine and industry.

## Graphical abstract

Stone motion during laser ablation is shown to result primarily from asymmetric cavitation bubble collapse, not from ejecta recoil. This challenges long‐standing assumptions and highlights the dominant role of bubble dynamics in laser–material interactions. The findings offer new strategies for improving precision in surgery, manufacturing, and other fluid–structure systems.

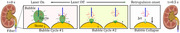

## 1 Introduction

Stone retropulsion – the undesired motion of the calculus away from the laser fiber during lithotripsy –remains a significant barrier to procedural efficiency, increasing treatment time and fiber placement accuracy. The predominant theory for stone retropulsion historically attributes this motion to the momentum transferred from the vaporized material (ejecta) expelled during ablation [1, 2, 3]. For the purpose of this work, all products of laser interaction with material, including vapor and solid particulates are collectively termed ejecta.

The observed correlations between retropulsion and factors such as pulse energy [4], crater volume [2, 4], stone mass [5], and pulse profile [2, 6] support this interpretation, based on experiments conducted with the fiber in contact with the stone. In line with this hypothesis, the observed increase in retropulsion for short‐pulses (150 μs) in Ho:YAG lasers may be attributed to higher peak power [7], which generates more forceful material ejection. In contrast, longer pulses with lower peak power reduce bubble pressure [8] and water streaming [9], leading to less stone displacement [7]. Further, the attenuation of retropulsion in water compared to air due to increased hydrodynamic drag and added mass [3, 4], is further strong evidence supporting this theory. However, exceptions remain: smaller‐diameter fibers with higher fluence have been shown to produce lower retropulsion‐to‐crater volume ratios [2], likely due to geometric differences in crater shape that influence the net ejecta momentum direction [3].

This ejecta‐centric paradigm does not fully capture the underlying physics–particularly in non‐contact scenarios or when ablation yield is low. Under these conditions, vapor bubble dynamics emerge as a potentially dominant contributor to stone motion. While previously treated as a secondary or peripheral effect–largely limited to microjet‐induced rocking motions [3, 6, 10]–asymmetric bubble collapse is a well‐documented propulsion mechanism in fluid‐structure interaction literature [11, 12, 13]. Traditionally, the vapor bubble generated during laser lithotripsy has been viewed primarily as a passive conduit for energy delivery to the stone surface–acting like a vapor tunnel, as in the MOSES effect [14]–rather than as a dynamic mechanical agent. However, this mechanism underpins applications ranging from ultrasonic cleaning [15, 16] and targeted drug delivery [17], to focused ultrasound therapies [18] and micromotors powered by cavitation impulse [19, 20].

In lithotripsy, these bubble‐driven impulses may be amplified or modulated by the presence and configuration of laser‐generated ejecta. Despite these possibilities, limitations in previous studies have restricted mechanistic insight. Most experiments employed contact‐mode fiber delivery, precluding precise control of fiber–stone distance–an essential parameter in bubble evolution [6]. In addition, high‐speed imaging that simultaneously captures bubble and stone dynamics is scarce. While Mohammadzadeh et al. [21] used acrylic phantoms to show bubble‐driven stone motion at standoff distances (SDs) >1 mm, their findings remain an exception. Other studies used interframe times ≥2 ms [3, 7], low‐frequency PIV [22], or thermal imaging–none of which are adequate for resolving microsecond‐scale cavitation events. Notably, no direct measurements of ejecta velocity have been reported in prior literature, making it difficult to quantitatively separate ejecta and bubble contributions.

Our study addresses these gaps by systematically examining the relative contributions of ejecta and bubble dynamics across varying SDs. We use Begostone phantoms [23] that are essentially freely suspended (See Section S1: Experimental Set‐Up, Stone preparation protocol, etc., and Figures S1–S4) to eliminate artifacts from mechanical support. Laser pulses were delivered individually with ample recovery time, allowing the stone to reset to its initial position. As successive pulses cause progressive material loss and reduced ablation yield (measured via optical coherence tomography, OCT), this experimental design enables us to decouple and isolate the respective contributions of ejecta and vapor bubble dynamics to retropulsion.

The results from this study provide new insights into laser‐fluid‐solid interactions and have implications for optimizing lithotripsy strategies, including SD selection, pulse shaping, and fiber placement. These findings establish a new physical interpretation: stone retropulsion arises primarily from bubble‐collapse dynamics modulated by ejecta, rather than from ejecta recoil itself.

## 2 Results and Discussion

### 2.1 Experiments in Water and Air at SD = 0.5 mm

To illustrate the fundamental dynamics of stone motion, we first examine the case of a 0.5 mm stand‐off distance (SD), representative of near‐contact laser lithotripsy conditions. This small separation is consistent with clinical practice, where the laser fiber typically operates in a partially contacted (defocused) configuration after the initial crater formation. A 365 μm fiber positioned 0.5 mm from a spherical Begostone phantom of 3 mm radius ($R_{st}$) was irradiated with a single short‐pulse (SP) or long‐pulse (LP) emission from a clinical Ho:YAG laser at an energy of 0.8 J.

**FIGURE 1:**
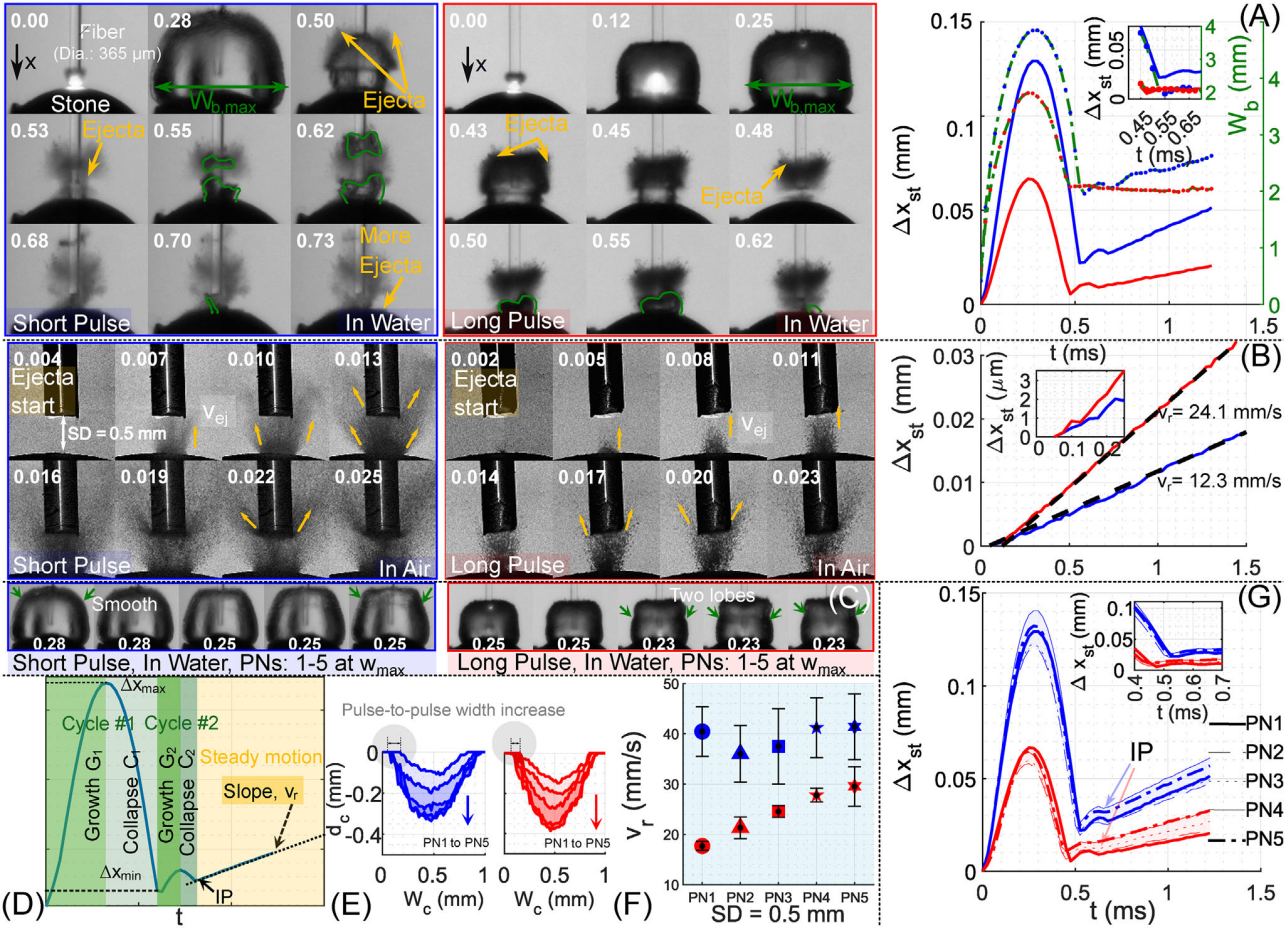
Retropulsion measurements in water and air for SD = 0.5 mm. The fiber diameter is 365 μm, and the dark curved region at the bottom corresponds to the stone, whose radius ($R_{st}$) is 3 mm. (A) Bubble images for SP (blue) and LP (red) at different time instants (in *ms*). Stone displacement ($\Delta x_{st}$) and bubble width evolution ($W_{b}$, shown in green with corresponding markers for SP and LP) were extracted using a custom MATLAB script. A thin green outline is added to distinguish the bubble from the ejecta (highlighted in yellow). The maximum bubble width, $W_{b,\max}$, is indicated. Note briefly, the displacement of the stone is too small to be readily discerned in individual time‐resolved frames. To enhance visualization, Figure S2 presents a streak image constructed by extracting a single pixel column (centered on the stone) from each frame and appending these columns sequentially in time, forming a synthetic space‐time diagram. (B) Selected ultra‐high‐speed camera images of a wet stone *in air* at different time instants (in *ms*) for SP and LP. Corresponding $\Delta x_{st}$ vs. time (t) plots appear to the right. The inset shows a lag in stone motion during the early time window. (C) A representative stone displacement trace illustrating key features: cycles of bubble growth (G) and collapse (C), and the inflection point (IP) at which the stone transitions to steady retropulsion with velocity $v_{r}$. The peak displacement during the first growth phase ($G_{1}$) is denoted $\Delta x_{\max}$, while the minimum during the first collapse phase ($C_{1}$) is $\Delta x_{\min}$. (D) Bubble shapes at their maximum width (shown in *ms*) across different pulse numbers (PN). Bubbles during initial pulses with more ejecta are more spherical; LP conditions produce two‐lobed bubble shapes in later pulses. (E) OCT‐measured crater depth ($d_{c}$) and width ($W_{c}$) as a function of PN for SP (blue) and LP (red). (F) Variation of retropulsion velocity $v_{r}$ with PN. p < 0.05 for each successive pulse comparison in the long‐pulse (LP) series except for the final interval, where p > 0.05, and no significant differences (p > 0.05) were found for the short‐pulse (SP) series. (G) Stone displacement $\Delta x_{st}$ across pulses PN 1–5 for SP and LP. Error bars denote the standard deviation from four or more replicates.

As shown in Figure 1A, stone motion occurred in synchrony with the primary bubble expansion and collapse, with no detectable lag (temporal resolution: 25 μs). When the same experiment was performed in air using a wet stone (Figure 1B), a similar net displacement was observed, but with a delayed (about 0.1 *ms*) initial response and no oscillatory transients–suggesting that cavitation dynamics in water play a more complex and significant role. Consistent with previous observations [3], ejecta initially exited nearly perpendicular to the surface (first 4 μs in Figure 1B), impacted the fiber tip, and then changed direction. The recorded images were processed using Lanczos3 interpolation (10× upscaling) to achieve an effective spatial resolution of ≈3μm/pixel. Sub‐pixel centroid tracking and temporal interpolation of the stone's region of interest enabled accurate displacement measurement down to approximately 1 μm. Stone displacement $\Delta x_{st}$ of 1 μm (interpolated) was reached after 159±22μs (SP) and 162±21μs (LP) from laser onset. Material ejection was observed until 150 μs (SP) and 250 μs (LP), consistent with their respective pulse durations.

The volume of ejecta in air, inferred from OCT‐measured crater volumes ($V_{c}$), was smaller for SP (0.015±0.002 mm^3^) than for LP (0.036±0.004 mm^3^, Table S1), whereas the corresponding ejecta velocities ($v_{ej}$) were 121±20 mm/s and 57±15 mm/s, respectively (See methodology in Figure S5). The retropulsion velocity, $v_{r}$–defined as the slope of the steady‐state stone displacement curve after the initial transients subside (Figure 1B)–was measured to be 12.3±1.3 mm/s for SP and 19.9±3.3 mm/s for LP. Although SP generated faster ejecta, its lower overall mass loss and more dispersed angular distribution of ejecta vectors [3, 4] produced a smaller net momentum transfer, resulting in a lower $v_{r}$, which could explain the lower $v_{r}$ in SP–consistent with expectations from the recoil momentum hypothesis. However, in water, the LP $V_{c}$ was 50% lower than in air (See Table S1), yet $v_{r}$ remained largely unchanged, challenging the expected correlation between $v_{r}$ and recoil momentum associated with ablated ejecta. Ultra‐high‐speed imaging at 5 Mfps (See Figure S6 and Movie S1) revealed discrete ejecta spurts lasting ≈40μs, with a consistent speed of $v_{ej}=113.0\;\pm \;16.1$ mm/s–nearly double that observed for wet stones in air, suggesting a different material removal mechanism in water that may involve a combination of photothermal ablation and micro‐explosions [24, 25]. Yet, this higher ejecta velocity did not translate to increased stone motion, particularly when accounting for the added mass effect in water, which should dampen recoil‐momentum generated motion. Even more striking was the result for SP pulses: $V_{c}$ in water were 15% lower than for wet stone in air (See Table S1), yet $v_{r}$ increased by 228%. Although we could not obtain clear images for this condition, even if a similar doubling of $v_{ej}$ were to be assumed for SP in water, such a dramatic rise in $v_{r}$ cannot be reconciled with the recoil momentum framework. Collectively, these findings suggest that ejecta momentum alone is insufficient to explain the observed stone motion, especially in aqueous environments.

The stone displacement ($\Delta x_{st}$) extracted from the high‐speed image sequences is shown in Figure 1A and summarized schematically in Figure 1D. During the initial bubble growth phase ($G_{1}$), the stone moves away from the fiber, reaching a maximum displacement ($\Delta x_{\max}$) of 120 μm at 0.28 ms for SP and 50 μm at 0.25ms for LP. As the bubble subsequently collapses ($C_{1}$), the stone reverses direction, returning toward the fiber with a minimum displacement ($\Delta x_{\min}$) of 20 μm for SP and 8 μm for LP. A secondary outward motion ($G_{2}$), driven by the rebound bubble, follows at 0.55 ms for SP and 0.50 ms for LP, after which the stone again shifts inward ($C_{2}$). The transition to a sustained outward motion–marking the inflection point (IP)–occurs at 0.7 ms for SP and 0.62 ms for LP, beyond which the stone exits the influence zone of the bubble and achieves a steady retropulsion velocity, $v_{r}$, measured as 40.45±4.9 mm/s for SP and 17.68±0.87 mm/s for LP. Notably, the number of oscillatory cycles preceding the IP–typically ranging from 1 to 3–is strongly influenced by both the characteristics of the bubble (e.g., maximum radius) and its proximity to the stone surface (see §3.4.2 [26]), as shown schematically for 2 cycles in Figure 1D. Across all cases, the IP consistently occurs during or near the end of the final collapse phase, reinforcing the dominant role of cavitation collapse in driving stone retropulsion.

A closer examination of the bubble dynamics for pulse number 1 (PN1) in Figure 1A reveals additional insight into the role of ejecta. After both the bubble and the stone reach their maximum extents, ejecta are observed puncturing the bubble boundary at approximately 0.5 ms for SP and 0.43 ms for LP (See Movies S2 and S3). The distinct optical contrast and straight, ballistic trajectories of the observed features confirm they are solid ejecta rather than microbubbles, which would display oscillatory and diffuse behavior. Their persistence and sharp edges across consecutive frames further indicate particulate rather than vapor‐phase motion (See Movies S4 and S5).

Notably, for SP, a secondary burst of ejecta [27] appears between 0.53 and 0.73 ms–well after the first bubble collapse $C_{1}$–whereas for LP, all visible ejecta are already expelled by the end of $C_{1}$ at 0.62 ms. The initial SP ejecta clearly originate from direct laser ablation, while the delayed second release could stem from residual material within the crater or from additional stone damage triggered by bubble collapse. This is supported by OCT‐based crater width measurements ($W_{c}$), which increase more prominently with PNs for SP than LP (Figure 1E), consistent with additional stone damage induced by bubble collapse. Crucially, no corresponding jumps or slope changes in the stone displacement ($\Delta x_{st}$) are observed at the times of this secondary ejecta release–either immediately or after accounting for possible inertial lag. This lack of kinematic response suggests that the ejecta, despite being present, do not contribute significantly to the net momentum transfer driving retropulsion, casting further doubt on the ejecta‐recoil hypothesis.

Concurrently, the stone moves away from the fiber during bubble expansion, and then reverses direction and moves toward the fiber during collapse (Figure 1A). At the IP, the net momentum transferred to the stone becomes sufficient to reverse its inward motion (velocity $v_{c}$) and propel it outward, effectively driving it away from the bubble's region of influence. This transition indicates that bubble collapse dynamics–notably the formation of high‐speed liquid microjets and residual flow structures such as ring vortices–are critical to the momentum exchange process [21, 28]. We therefore propose that it is the impulse delivered by these collapse‐induced flows at IP that dominates the onset of sustained retropulsion.

While the directional change at the IP could, in principle, result from recoil momentum imparted by ejecta, our findings suggest otherwise. To isolate the role of ejecta, we examined a sequence of five laser pulses delivered to the same stone, during which the ejecta yield progressively diminished (Figure S7). As the ejecta burden decreased, the corresponding bubble shapes for both SP and LP became increasingly asymmetric and less spherical at their maximum widths ($W_{b,\max}$), as seen in the image panel, Figure 1C. For the purposes of this work, we consider the bubble radius, $R_{max}$ to be 0.5$\times \;W_{b,\max}$.

High‐speed imaging of wet stone in air further revealed that ejecta initially impact the fiber and then migrate toward the upper portion of the bubble (Figure 1B), likely altering the local energy deposition and resulting in more curved bubble shape at the top for the initial pulses. As the crater evolves with successive pulses (PNs), both the crater dimensions and the resulting $v_{r}$ vary, as shown in Figure 1E,F. The corresponding stone displacement trajectories for SP and LP are shown in Figure 1G.

Importantly, we observe that $\Delta x_{\min}$–the inward displacement during the first collapse–systematically increases with PNs, despite a clear reduction in ejecta volume. This inverse relationship suggests that the stone's motion cannot be solely attributed to ejecta recoil. Instead, the data point toward a growing contribution from bubble collapse dynamics, as will be elaborated in the subsequent sections.

**FIGURE 2:**
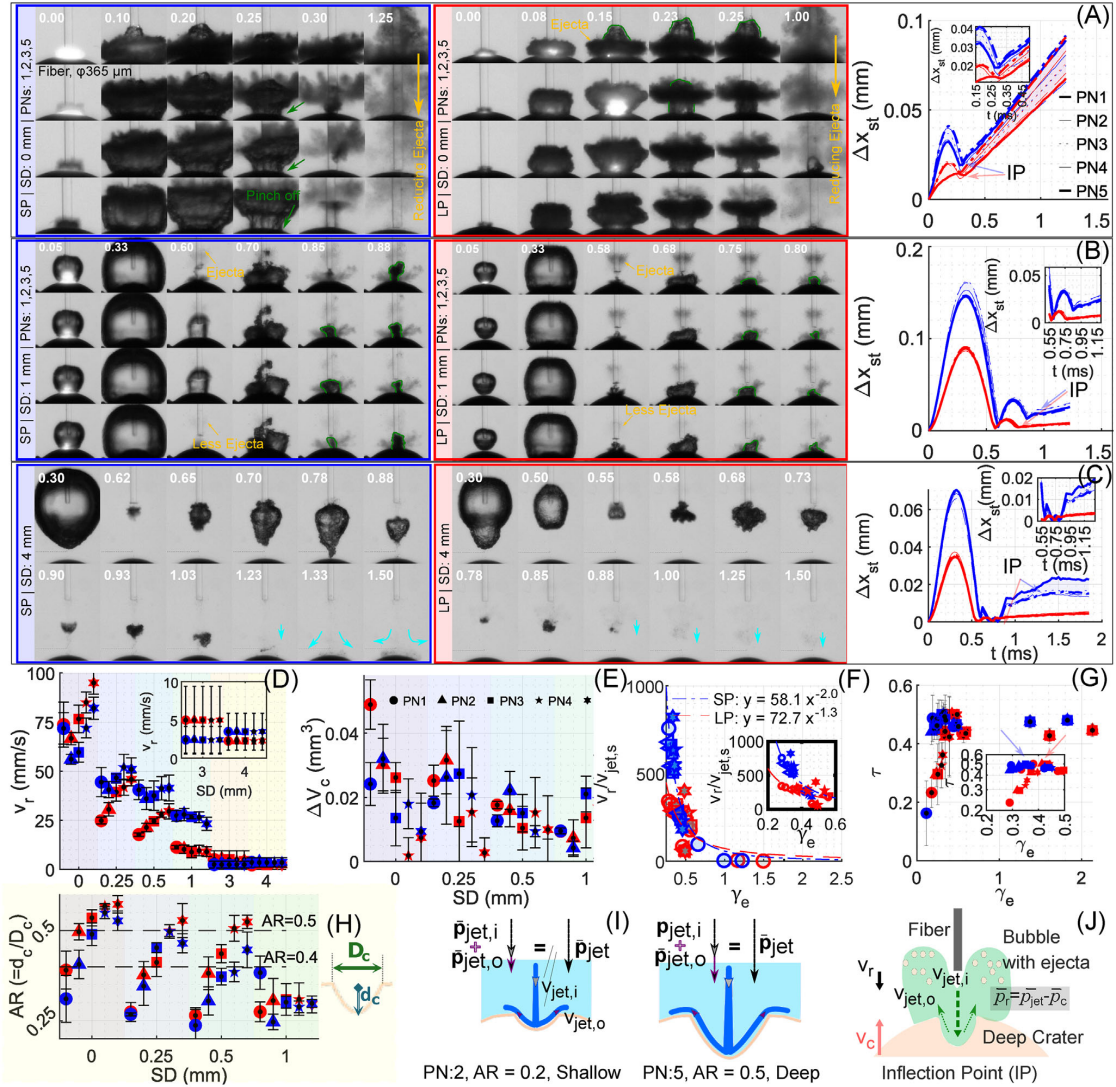
Retropulsion in water for different SDs with short (SP, blue, FWHM = 80 μs) and long (LP, red, FWHM = 205 μs) pulses. (A–C) Bubble images at SD = 0, 1, 4 mm for PN1–PN5; bubble shapes vary slightly due to decreasing ejecta. Stone displacement data (with insets at inflection point, IP) shown to the right. Bubble contours (green), ejecta (yellow), and vortical flow (cyan). (D) Retropulsion velocity $v_{r}$ vs. SD; inset: SD = 3–4 mm. Tabulated values of $v_{r}$ and statistical comparisons between consecutive pulses, as well as vs. PN1, are reported in Tables S3 and S4. (E) Crater volume change $\Delta V_{c}$ decreases with PN. (F) (F) Retropulsion velocity $v_{r}$ scaled by the jet velocity scaling factor $v_{\text{jet},s}\propto SD_{e}^{2}/R_{\max}^{5/2}$ is plotted against the effective standoff distance, $\gamma_{e}=SD_{e}/R_{\max}$, where $SD_{e}=SD+d_{c}$. The scaled relationship follows a power law: $v_{r}/v_{\text{jet},s}=33.7\;\gamma_{e}^{-2.5}$ ($R^{2}=0.99$) for SP and $32.8\;\gamma_{e}^{-2.1}$ ($R^{2}=0.79$) for LP. All data points scale well, except for the LP case at SD = 0 mm, possibly due to scaling limitations under strong geometric confinement, where the bubble cannot expand freely due to the narrow gap between the fiber tip and the stone surface. (G) Scaled time $\tau =t_{c}/\left(t_{g}+t_{c}\right)$ vs. $\gamma_{e}$: peaks at $\gamma_{e}∼0.35$ (SP), ∼0.4 (LP). (H) Crater aspect ratio AR $=d_{c}/D_{c}$ vs. SD: SP plateaus; LP rises linearly from 0.25–0.5 mm. Inset defines $d_{c}$, $D_{c}$. (I) Schematic illustrating how jet confinement varies for shallow vs. deep craters, resulting in greater net jet momentum ${\bar{p}}_{\text{jet}}$ in the latter case. (J) Schematic illustrating the proposed hypothesis for retropulsion in water. Here, $v_{\text{jet},i}$ and $v_{\text{jet},o}$ are the jet velocities entering and exiting the crater. The total stone momentum is a vector sum of ${\bar{p}}_{c}$ (momentum at IP) and ${\bar{p}}_{\text{jet}}$. *Note*: x‐axes show SD, not $SD_{e}$. Values at each marker = PN1–PN5. Plot layout matches Figure 1F, shaded by SD groups (0, 0.25, 0.5, 1, 3, 4 mm). Error bars indicate standard deviation across replicates.

A particularly intriguing observation is the increasing trend of $v_{r}$ with PNs, rising by 2.3% for SP and a striking 67% for LP (Figure 1F). This finding is counterintuitive under the conventional recoil momentum framework. As PN increases, the ejecta mass decreases (Figure 1E, also Figure 2E), and the ejecta velocity ($v_{ej}$) diminishes due to reduced laser fluence and deepening crater depth ($d_{c}$, Figure S8). Additionally, the evolving crater geometry causes ejecta vectors to diverge, partially canceling each other and further diminishing net recoil momentum (${\bar{p}}_{r}$).

Even when considered through the lens of bubble collapse theory, this increasing $v_{r}$ with PN remains unexpected. Elongated bubble shapes oriented perpendicular to the surface (as seen in Figure 1C) are known to reduce the velocity of collapse jets and the associated impact pressure (see §3.5 of [26]), which would typically lead to lower $v_{r}$.

We therefore propose an alternative explanation: the evolving crater geometry (Figure 1E) may enhance the coupling efficiency in momentum transfer between the collapse jet and the stone. Rather than pressure‐driven forces alone, it is the directed jet momentum (${\bar{p}}_{\text{jet}}$) and its interaction with the modified crater surface that likely dominate the retropulsion dynamics. In the next section, we explore how varying the SDs influences these dynamics.

### 2.2 Experiments With Varying SDs

#### 2.2.1 Contact‐Mode Regime (SD = 0 mm)

Figure 2A shows bubble images and stone displacement plots for PNs 1, 2, 3, and 5. As the ejecta diminishes with increasing PN (see frames at 1.25 ms for SP and 1 ms for LP), the emerging bubble shape becomes progressively clearer, revealing a strong one‐to‐one correlation between ejecta‐modulated bubble morphology and resulting stone motion. This trend highlights our central hypothesis: ejecta sculpts the bubble, and the bubble collapse primarily drives the retropulsion. As shown in Figure 2D, $v_{r}$ increases by 14% for SP (from 71.6 ± 8.0 mm/s to 81.6 ± 5.6 mm/s) and by 28.7% for LP (from 73.7 ± 11.4 mm/s to 94.9 ± 5.8 mm/s) with PNs. The LP exhibits slightly higher $v_{r}$ at this SD, contrary to previous literature [2, 6], likely due to improved standoff control and reduced experimental variability in our study. Notably, the enhanced retropulsion with reducing ejecta in LP, further favors the notion that lower amount of ejecta, a modified bubble shape, and collapse dynamics due to the presence of a deeper craters, all play a role in stone retropulsion.

In this contact‐mode regime, bubble expansion is constrained by the proximity of the fiber to the stone, leading to a rapid collapse ($t_{C}$ = ∼70 μs). Consequently, the jet momentum ${\bar{p}}_{\text{jet}}$ imparted at the IP readily overcomes the opposing collapse‐acquired momentum (${\bar{p}}_{c}$), producing a net motion of the stone away from the fiber and resulting in high $v_{r}$ at this SD (see illustration in Figure 2J). The consistent occurrence of the IP at the end of the first cycle minimizes energy dissipation and maximizes momentum transfer, further contributing to the higher $v_{r}$ values observed for SD = 0 mm (Figure 2D). Importantly, the increase in $v_{r}$ with successive pulses (PNs) therefore arises not from ejecta recoil, but from enhanced hydrodynamic coupling between the evolving crater and the collapse jet, as the crater depth and curvature progressively shape the bubble's asymmetric collapse (Figure 2I). This observation directly supports our central hypothesis that ejecta‐modulated bubble dynamics, rather than recoil from ablation products, governs stone retropulsion. It is worth noting that at SD = 0.25 mm (see Figure S9) and 0.5 mm (see Figure 1), the inflection point consistently shifts to the end of the second cycle, indicating a transition in momentum exchange dynamics as SD increases.

#### 2.2.2 Non‐Ablative Regime (SD $\geq \;R_{max}$)

For SDs where ablation does not occur (Figure 2C), stone motion enters a distinct non‐ablation‐driven regime. At these larger distances (SD ≥ $R_{max}$), the laser pulse energy is confined within the bubble, preventing any material removal. Despite this, the stone still undergoes oscillatory push–pull motion driven by the nearby bubble dynamics. In this regime, the location and symmetry of bubble collapse–rather than ejecta or jet impact–become the dominant factors governing the IP. For example, in some LP cases at SD = 3 mm (see Figure S10), the rebound bubble collapses toward the fiber, producing a weak “net suction” that steadily pulls the stone toward the tip. This motion, opposite to conventional retropulsion, is consistent with re‐entrant inflow phenomena previously reported during Ho:YAG lithotripsy. Fried et al. [25] described similar transient flow reversals and localized suction arising from micro‐explosions and water absorption near the fiber tip, while Lee et al. [4] attributed them to re‐entrant water flow during post‐collapse refilling of water toward the cavity, producing a transient negative‐pressure region capable of drawing detached particles or nearby surfaces toward the laser source. These flow reversals closely resemble synthetic‐jet formation driven by repeated cavitation collapse, as demonstrated by Mohammadzadeh et al. [21], where alternating vortex‐pair dynamics produce a steady jet and accompanying suction near the boundary.

In contrast, as shown in Figure 2C, for SP at SD = 4 mm, the second bubble cycle peaks at approximately 0.78 ms, but the bubble remains far from the stone surface. This leads to a brief period of quiescence, after which the third collapse–occurring closer to the stone (see §1 of [26])–produces vortical flows (from 1.23 ms onward, marked in cyan) that finally retropulse the stone (displacement occurring at 3.5±2.4 ms). For LP at the same SD, the more elongated bubble shape causes the collapse to occur even farther from the solid boundary (see 0.68 ms for LP vs. 0.78 ms for SP), generating a much weaker impulse on the stone and resulting in an even lower retropulsion velocity $v_{r}=2.2\;\pm \;1.7$ mm/s.

These findings emphasize that even in the absence of ablation, retropulsion is possible, though significantly delayed and weaker, arising only when the collapse zone of the bubble sufficiently overlaps with the stone. Altogether, these observations highlight the critical role of bubble collapse proximity and asymmetry, even without ejecta, in driving retropulsion–thus extending the relevance of our central hypothesis beyond just ablation‐dominant regimes.

#### 2.2.3 Ejecta‐Modulated Regime: SD = 1 mm

While SD = 0.25 mm and 0.5 mm show nearly comparable retropulsion behavior, SD = 1 mm reveals a distinctive shift in the $v_{r}$ trend with PN (Figure 2D), attributable to reduced ejecta load and its influence on bubble evolution. At this SD (Figure 2B), the combination of a relatively strong bubble collapse and a larger contact area between the stone and fluid results in the maximum value of $\Delta x_{\max}$ (Figure S11a). Specifically, $\Delta x_{\max}$ for the SP is 2.5× higher than at SD = 0 mm (0.08±0.004 mm), and 5.3× higher for the LP, reaching 0.09±0.003 mm.

Despite this large $\Delta x_{max}$ and favorable bubble size ($v_{jet,i}$ ∝ $R_{max}$), the stone undergoes three distinct damping cycles of oscillatory motion–alternating between bubble‐driven repulsion and suction–before reaching the IP, where it is retropulsed. The resulting retropulsion velocities are $v_{r}=27.30\pm 2.8$ mm/s for SP and 11.24±0.9 mm/s for LP. These values are significantly lower than what might be expected based on the large $\Delta x_{\max}$ or the inferred jet velocity $v_{\text{jet},i}$, suggesting that energy dissipation over multiple cycles reduces the effective momentum transfer during jet‐stone interaction.

Moreover, while crater depth ($d_{c}$) and surface area ($A_{c}$) increase with PN (see Figure S8, and crater volume ($V_{c}$) change decreases (Figure 2E), the $v_{r}$ either remains constant (LP) or declines (SP). This decoupling between ablation metrics and $v_{r}$ provides further evidence that ejecta modulates bubble morphology, crater shape and subsequent jet formation, which in turn governs retropulsion–rather than direct momentum from ejected mass.

### 2.3 Scaling Behavior in Laser‐Stone Interactions

To further understand how bubble dynamics drive stone retropulsion, we explored a scaling framework based on the jet velocity hypothesis, wherein the magnitude of stone motion is primarily dictated by the momentum imparted by the collapsing jet. Using a jet velocity expression adapted from Outi et al. [29], we defined a representative jet speed, $v_{jet,s}$ ∝ $\frac{SD_{e}^{2}}{{R_{max}^{*}}^{5/2}}$, where the effective standoff distance $SD_{e}=SD+d_{c}$ accounts for both the SD and the growing crater depth ($d_{c}$). A correction factor was included to $R_{max}$ based on the curvature of the stone to obtain $R_{max}^{*}$, details of which may be found in Section S4. The normalized standoff distance, $\gamma_{e}$ (= $\frac{SD_{e}}{R_{max}}$), was used to non‐dimensionalize the data. As shown in Figure 2F, this scaling effectively collapsed the SP data ($R^{2}=0.88$), indicating a strong correlation between $v_{r}$ and $v_{\text{jet,s}}$. However, the LP data showed weaker agreement ($R^{2}=0.49$), possibly due to the greater sensitivity of long‐pulse bubbles to ejecta‐driven shape distortions, which this scaling does not capture.

Recognizing that events during bubble collapse, and not just during growth, are central to stone acceleration, we examined bubble growth ($t_{g}$) and collapse durations ($t_{c}$). Despite the temporal resolution limitation of our camera at 25 μs, subtle differences were observed between SP and LP cases (Figure 2B), which were attributed to the presence or absence of internal ejecta modulating the collapse behavior (see Figure S12a). Following an earlier work on spherical particle interaction with bubbles [30], a scaling factor for bubble time, defined as τ =$t_{c}/\left(t_{c}+t_{g}\right)$, was plotted against $\gamma_{e}$ in Figure 2G. This parameter τ, which describes the dominance of the collapse time relative to total bubble life ($t_{c}+t_{g}$), peaked at SD = 0.25 mm for SP and SD = 0.5 mm for LP–distances at which retropulsion enhancement was also maximized.

This observation highlights the coupled influence of bubble growth dynamics ($t_{g}$), collapse duration ($t_{c}$), and effective standoff distance ($SD_{e}$), which together control the jet momentum. The SP condition at SD = 0.25 mm shows enhanced $v_{r}$, while at 0.5 mm the increase is minor (2.3%), reflecting the sensitivity of jet impulse to confinement (also see Figure S12c). Notably, a demarcation in τ behavior in Figure 2G occurs around $\gamma_{e}\approx 1$, implying the existence of two distinct regimes: a jet‐dominated regime at $\gamma_{e}<1$, and an inertia‐dominated regime at $\gamma_{e}>1$, consistent with prior studies [30].

We hypothesized that crater geometry, particularly its aspect ratio (depth‐to‐width), could further influence jet focusing and coupling efficiency in this jet‐dominated regime. Figure 2H shows how the crater aspect ratio varies with SD and PN, while Figure 2I highlights the increasing trend in jet momentum coupling. These findings indicate that crater evolution and ejecta‐modulated bubble asymmetry enhance the efficiency of jet–stone momentum transfer during collapse, a mechanism not captured by conventional recoil or single‐parameter jet‐speed models.

To integrate these interdependent factors–standoff geometry, bubble dynamics, and evolving crater morphology – we conducted a multivariate regression analysis (see details in Section S6), revealing that ejecta‐driven modifications to bubble shape and collapse symmetry are central to stone retropulsion.

### 2.4 Multivariable Scaling Analysis of Ejecta‐Modulated Bubble‐Induced Stone Motion

To test the hypothesis that stone retropulsion is primarily governed by cavitation bubble collapse modulated by ejecta and crater geometry, we developed a dimensionally consistent multivariable scaling model starting from the principle of dimensional homogeneity using the Buckingham Pi theorem [31]. The predicted retropulsion velocity $v_{r,p}$ is expressed in a dimensionless form of geometric, temporal, and morphological parameters normalized by a reference collapse velocity $v_{\text{ref}}$, and expressed as:

(1) $$\begin{array}{cc} \frac{v_{\text{r,p}}}{v_{\text{ref}}} & =C{\left(\frac{SD_{e}}{R_{\max}}\right)}^{\alpha }{\left(\frac{t_{c}}{t_{g}+t_{c}}\right)}^{\beta }\\ & \;\times {\left(1+\frac{R_{\max}}{d_{c}}\right)}^{\eta }{\left(1+\frac{d_{c}}{D_{c}}\right)}^{\kappa }{\left(1+\frac{A_{c}}{SD_{e}^{2}}\right)}^{\zeta } \end{array}$$

Here, C is a dimensionless empirical coefficient obtained by regression. Equation (1) provides a dimensionally homogeneous scaling model with clear physical interpretation (see Section S6) that accurately predicts retropulsion velocity within the experimentally validated parameter regime; however, it is not intended as a universally applicable predictive tool beyond this range. The reference velocity $v_{ref}=\sqrt{(}p_{\infty }/\rho_{w})$ was derived from the inertial balance in the Rayleigh–Plesset equation, representing the characteristic collapse velocity of a bubble in water driven by a 1 atm pressure difference [32]. Substituting $p_{\infty }=1.013\times \;10^{5}\mathrm{Pa}$ and $\rho_{w}=1000\;kg/m^{3}$ yields $v_{ref}=10.07\;m/s$. This normalization is standard in cavitation collapse analyses. The variables $SD_{e}$, $t_{c}$, $t_{g}$, $R_{\max}$, $d_{c}$, $D_{c}$, and $A_{c}$ represent the geometric and temporal parameters influencing bubble–stone interactions. The exponents α,β,η,κ, and ζ were determined through regression analysis using collective data from both SP and LP experiments across all PNs and SDs. Each exponent quantifies the relative physical contribution of its corresponding term to the momentum transfer from bubble collapse to stone motion, as modulated by the evolving ejecta and crater geometry.

#### 2.4.1 Role of Crater Evolution and Ejecta‐Modulated Crater Geometry

Successive laser pulses progressively ablate material and reshape the stone surface, gradually deepening craters and continuously modifying local boundary conditions for cavitation collapse. This creates a dynamic system where each pulse acts on surfaces already shaped by previous ejecta, altering subsequent jet direction [33], symmetry, and energy focusing characteristics of bubble collapses.

The $SD_{e}=SD+d_{c}$ incorporates crater depth $d_{c}$ and reflects how successive material loss progressively displaces the collapse region relative to the fiber tip. This affects both the spatial configuration of the bubble and jet‐stone coupling. Experimental data show that $d_{c}$ ranges from 0.13 to 0.42 mm for LPs and 0.11 to 0.40 mm for SPs, significantly modifying $SD_{e}$.

#### 2.4.2 Model Performance and Parameter Significance

**FIGURE 3:**
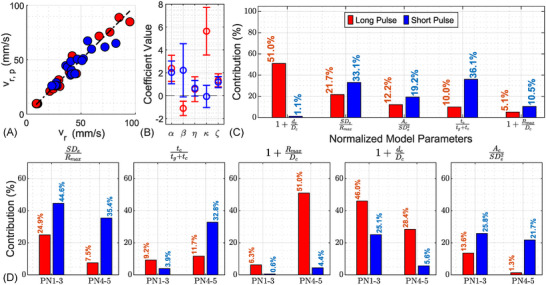
(A) Comparison between experimental retropulsion velocities and predictions from the multivariable scaling model, normalized by a reference collapse speed $v_{\text{ref}}\;=\;10\;\text{m/s}$, across all standoff distances (SDs) and pulse numbers (PNs). LP data points (blue): α=2.39, β=−1.11, η=0.56, κ=5.63, ζ=1.34, $C_{\text{LP}}=1.445\times 10^{-4}$, and $R^{2}=0.981$. For SP data points (red): α=2.03, β=2.22, η=0.65, κ=−0.07, ζ=1.18, $C_{\text{SP}}=2.504\times 10^{-2}$, and $R^{2}=0.864$, (B) Regression coefficient estimates with 95% confidence intervals for LP (blue) and SP (red), (C) Comparative percentage contributions of different dimensionless model parameters to predicted stone retropulsion velocity for LP and SP across all PNs. (D) Dimensionless Parameter Sensitivity with Pulse Progression for LP and SP.

As shown in Figure 3A, the model accurately predicts the normalized retropulsion velocity, $v_{r,p}/v_{\text{ref}}$, with data points tightly aligned along the 1:1 line. The model explains 98.1% of the variance in LP data and 86.3% in SP data. Deviations from the diagonal are minimal, suggesting low bias and good overall model fit. The residual analysis (see Figure S13) shows no systematic bias or heteroscedasticity, confirming the statistical adequacy of the model. Figure 3B confirms that all regression coefficients, including temporal and geometric terms, are statistically significant.

For compact representation, the geometric terms are grouped as (see Section S6 for detailed representation):

$$\begin{array}{cc} G_{\text{LP}} & ={\left(1+\frac{R\max}{D_{c}}\right)}^{0.564}{\left(1+\frac{d_{c}}{D_{c}}\right)}^{5.63}{\left(1+\frac{\pi }{4}\cdot \frac{D_{c}^{2}}{SD_{e}^{2}}\right)}^{1.34}\;\\ G_{\text{SP}} & ={\left(1+\frac{d_{c}}{D_{c}}\right)}^{-0.07}{\left(1+\frac{\pi }{4}\cdot \frac{D_{c}^{2}}{SD_{e}^{2}}\right)}^{1.18}{\left(1+\frac{R_{\max}}{D_{c}}\right)}^{0.65} \end{array}$$

Then, with $\tau =\frac{t_{c}}{t_{g}+t_{c}}$, predictive model simplifies to:

$$\begin{array}{cc} \frac{v_{\text{r,p,LP}}}{v_{\text{ref}}} & =\frac{C_{\text{LP}}\cdot G_{\text{LP}}}{SD_{e}^{2.39}\cdot R_{\max}^{1.00}\cdot \tau^{1.11}}\\ \frac{v_{\text{r,p,SP}}}{v_{\text{ref}}} & =\frac{C_{\text{SP}}\cdot \tau^{2.22}}{SD_{e}^{2.03}\cdot R_{\max}^{1.00}\cdot G_{\text{SP}}} \end{array}$$

where, the dimensionless constants were computed as: $C_{\text{LP}}=1.445\times 10^{-4}$ and $C_{\text{SP}}=2.504\times 10^{-2}$.

#### 2.4.3 Physical Interpretation of Model Parameters

The regression analysis reveals distinct physical mechanisms driving stone retropulsion under LP and SP conditions, as reflected in Figure 3A–C.

The term ${\left(\frac{SD_{e}}{R_{\max}}\right)}^{\alpha }$ captures the geometric relationship between ${\mathrm{SD}}_{e}$ and the maximum bubble radius, reflecting the geometric proximity of the fiber tip to the bubble. This contributes 33.1% in SP and 21.7% in LP (see Section S6.2 for details on the methods used to calculate these percentages). The higher exponent in LP (α=2.39 vs. α=2.03 in SP) suggests that fiber positioning becomes increasingly critical as crater geometry evolves, reinforcing the cumulative influence of successive pulses in LP treatments.

The measure of energy concentration during bubble collapse is represented by collapse ratio $\tau =\frac{t_{c}}{t_{g}+t_{c}}$. Here, bubble growth time ($t_{g}$) demonstrates the vaporization‐driven expansion phase, during which energy accumulates as potential energy, approximated by $E_{b}=\frac{4}{3}\pi R_{max}^{3}\left(p_{\infty }-p_{b}\right)$ for an equivalent spherical bubble. The collapse time ($t_{c}$), governed by the pressure differential $\left(p_{\infty }-p_{b}\right)$, reflects the rate at which this stored energy is released. The term $\tau^{\beta }$ is most influential in SP, where it contributes 36.1% with a strong positive exponent β=2.22, underscoring the importance of rapid, impulsive collapse events in driving SP‐induced retropulsion. In contrast, LP exhibits a negative β=−1.11 and lower contribution (10.0%), suggesting that LP‐induced retropulsion is driven more by gradual energy accumulation and volumetric bubble growth shaped by prior ablation. The mild deviation of the LP data at nominal SD = 0 mm likely reflects normalization limitations in the strongly confined‐bubble regime, where restricted bubble growth and curvature‐induced jet modification (Tomita et al. [34]; Supponen et al. [29]) reduce the effective retropulsive impulse relative to model predictions.

The interaction between the bubble and the evolving crater geometry is influenced by the bubble‐to‐crater diameter ratio, represented by the term ${\left(1+\frac{R_{\max}}{D_{c}}\right)}^{\eta }$. This dimensionless parameter captures the degree of alignment between the collapsing bubble and the crater opening, which influences jet directionality and energy focusing, and it is designed to ensure physical continuity at vanishing crater depth ($d_{c}$ → 0), avoiding divergence in the regression model. It contributes modestly in both cases – 5.1% for LP and 10.5% for SP – with positive exponents η=0.56 (LP) and η=0.65 (SP). These values suggest that even limited alignment enhances jet‐induced momentum transfer. While LP benefits from well‐defined crater boundaries due to greater ablation, the positive η in SP indicates that early‐stage craters still provide some directional confinement, contributing to efficient energy delivery. Thus, this parameter plays a secondary but consistent role in enhancing retropulsion through geometric focusing.

The crater depth‐to‐diameter ratio ${\left(1+\frac{d_{c}}{D_{c}}\right)}^{\kappa }$ contributes 51.0% to the predictive model variance, with a high fitted exponent κ=5.63. This indicates a strong dependence on crater evolution, where ablation‐induced shape changes focus of the collapsing fluid axially toward the stone's center of mass, significantly amplifying retropulsion. In contrast, this parameter contributes just 1.1% in SP, with a near‐zero exponent (κ=−0.07), confirming that crater geometry plays a negligible role when collapse is highly impulsive and minimally shaped by surface evolution.

Finally, the contact area term ${\left(1+\frac{A_{c}}{SD_{e}^{2}}\right)}^{\zeta }$ grows in importance from 12.2% in LP to 19.2% in SP, suggesting a stronger surface‐coupling influence under SP, where collapse is sharper and more localized. The positive exponents for both LP (ζ=1.34) and SP (ζ=1.18) suggest that increased contact area enhances the momentum transfer during collapse, likely by improving jet coupling with the stone surface.

In summary, LP‐induced retropulsion is governed by geometrically focused bubble collapse modulated by crater evolution, while SP‐induced retropulsion is dominated by impulsive, temporally concentrated energy delivery and sensitivity to fiber–stone positioning. These contrasting parameter dependencies reflect two fundamentally different physical regimes of momentum transfer dictated by laser pulse duration.

Equation (1) provides a dimensionally consistent and validated description of the observed retropulsion behavior within the tested clinically relevant experimental data set range. Further validation with independent datasets and varied laser–stone conditions will be required in the future to establish predictive generality.

#### 2.4.4 Dimensionless Parameter Sensitivity With Pulse Progression

As shown in Figure 3D, the contribution of dimensionless model parameters evolves across PNs.

In early LPs (PN 1–3), $\left(1+\frac{d_{c}}{D_{c}}\right)$ is the dominant contributor, accounting for 46% of the model variance–substantially higher than the corresponding SP value of 25.1%. This highlights the rapid morphological evolution of the crater in LP experiments, where significant material removal during the initial pulses dramatically alters the boundary conditions, enhancing collapse asymmetry and increasing jet‐driven retropulsion.

However, the influence of $\left(1+\frac{d_{c}}{D_{c}}\right)$ diminishes to 28.4% during later LPs (PN 4–5), while the importance of $\left(1+\frac{R_{\max}}{D_{c}}\right)$ rises markedly to 51.0%. This shift suggests that as the crater widens, the spatial alignment between the bubble and the evolving crater opening becomes increasingly critical for directing collapse forces and maximizing momentum transfer to the stone.

For SPs, the relative contributions of $SD_{e}/R_{\max}$ and the $t_{c}/\left(t_{g}+t_{c}\right)$ remain high and consistent across PNs, reaffirming the importance of geometric proximity and impulsive energy concentration in SP‐induced retropulsion. Meanwhile, the influence of term $\left(A_{c}/SD_{e}^{2}\right)$ decreases slightly from 25.8% in PN 1–3 to 21.7% in PN 4–5, indicating a modest reduction in the role of surface coupling as crater geometry stabilizes.

Overall, these trends illustrate the evolving balance between crater geometry, bubble dynamics, and stone motion across PNs. Early pulses benefit most from optimized fiber–stone alignment and shallow boundary conditions, whereas later pulses require enhanced coupling between the bubble and the matured crater geometry to sustain efficient momentum transfer.

### 2.5 Our Hypothesis: Ejecta‐Modulated Bubble Collapse for ${\bar{p}}_{r}$:

As illustrated in Figure 2J, three primary factors govern stone retropulsion momentum (${\bar{p}}_{r}$): (i) the stone's instantaneous velocity at the IP just before collapse ($v_{c}$), (ii) the velocity of the liquid jet during collapse ($v_{jet,i}$), and (iii) the efficiency of momentum coupling between the jet and the stone (AR). Amongst these, $v_{c}$ and $v_{jet,i}$ are determined primarily by the spatial and temporal evolution of the bubble–its growth ($t_{g}$) and collapse times ($t_{c}$), maximum radius ($R_{max}$), and elongation of the bubble.

Furthermore, as the bubble undergoes more oscillatory cycles with increasing $\gamma =SD/R_{\max}$ before reaching its final collapse (see §3.4.2 of [26]), energy losses increase [35], resulting in a lower $v_{r}$ (Figure 2F). This interpretation is reinforced by ureteroscope experiments [36], where retropulsion was replaced by suction at certain offset distances when bubble collapse occurred away from the stone, highlighting that bubble dynamics exerts a more dominant influence on stone motion than ejecta alone.

#### 2.5.1 Boundary Shape, Crater Aspect Ratio and Jet Coupling Efficiency ‐ ${\bar{p}}_{\text{jet}}$:

As explained earlier, from the work of Vogel et al. [26], elongation of the bubble perpendicular to the surface reduces the velocity of the jet ($v_{\text{jet,i}}$) and the resulting pressure on the stone. In LP cases, deeper craters help offset reductions in $v_{\text{jet,i}}$ by enhancing coupling efficiency and the final jet momentum imparted to the stone, ${\bar{p}}_{\text{jet}}$. This increased jet confinement contributes to an increase in retropulsion velocity ($v_{r}$) with PN. In contrast, for SP, although the crater shape can provide partial confinement, the plateauing of AR (Figure 2J) suggests that it is likely insufficient to increase ${\bar{p}}_{\text{jet}}$ enough to steadily enhance ${\bar{p}}_{r}$ with PN as observed in the LP, particularly at intermediate SDs. For SDs <0.25 mm, however, since the craters tend to reach ARs ≥ 0.5, an increase in ${\bar{p}}_{r}$ (and hence, $v_{r}$, Figure 2D) with PN was observed. The role of AR is especially evident at SD = 1 mm, where both AR and $v_{r}$ remain flat or decrease with PN (see Figure 2D,H), confirming a direct correlation. This contribution arises from a change in the solid boundary that modifies the intensity of bubble collapse.

#### 2.5.2 Bubble Shape, and Stone Momentum at IP ‐ ${\bar{p}}_{c}$

To assess the contribution of recoil momentum from ejecta, we compared in‐air and underwater dynamics. As shown in Figure 1B, stone displacement in air is sluggish, where recoil is the only acting force. Taking into account the added mass effect in water (approximately 1.58 × greater inertia, see Section S7.2), retropulsion from ejecta should be even more delayed – now estimated to occur 252−256μs after ejecta emerge for SP and LP, respectively. Since the onset of ejecta occurs approximately 60μs [37] after the start of the laser pulse, recoil momentum contribution would be felt only during the suction phase ($C_{1}$, see Figure 1D), well after the stone has already begun reversing direction toward the fiber (at 0.280ms for SP, and 0.250ms for LP, see Figure 1A). With ejecta in water emerging in bursts (Figure S6), and the presence of hydrodynamic drag underwater, the response would only be delayed further. If recoil momentum did contribute significantly, it would be evident as a reduced inward displacement (i.e., higher $\Delta x_{min}$) during collapse. However, our results show the opposite trend: $\Delta x_{min}$ increases as ejecta diminishes (i.e., with increasing PN, Figure S11b, S11c). This claim is also supported by our numerical estimates and hydrophone measurements (see Section S5) of the pressure forces involved in retropulsion.

Since $\Delta x_{min}$ denotes the distance at which the stone halts its motion toward the fiber during collapse, the greater the distance from the fiber at which the stone stops (i.e., higher $\Delta x_{min}$), the lower the velocity $v_{c}$. Consequently, $\Delta x_{min}$ serves as a reliable proxy for $v_{c}$; a lower $|v_{c}|$ indicates that the stone is more susceptible to jet‐induced repulsion at the IP (Figure 2J). Therefore, a reduction in ${\bar{p}}_{c}$ increases ${\bar{p}}_{r}$, even without accounting for an increase in ${\bar{p}}_{\text{jet}}$, as previously discussed. This contribution stems solely from the bubble dynamics, altered by the decreasing ejecta, which could arise from combined mechanical and thermal effects (see Section S3.1, S3.2).

### 2.6 Clinical Implications

From a clinical perspective, the condition of SD = 0, corresponding to true contact between the fiber and the stone, is ideal for precision but is rarely maintained due to patient movement, respiratory motion, ureteral curvature, and retropulsion. Previous findings [38] indicate that maintaining a small, controlled near‐contact gap (approximately 0.25–0.50 mm) can be beneficial, as it activates vapor‐bubble collapse and microjet formation that significantly enhance per‐pulse material removal beyond pure photothermal ablation. The comparison demonstrated that cavitation could increase the effective ablation volume by 50–75% depending on SD, even outside strict contact. Although ejecta recoil can arise during the first contact pulse, subsequent pulses will occur in non‐contact conditions due to rapid crater formation. Therefore, clinically relevant retropulsion is governed primarily by bubble‐collapse impulses rather than ablation recoil. This transition from contact to non‐contact defines the ejecta‐modulated bubble regime that underpins this study, emphasizing that non‐contact SDs (SD > 0 mm) are most relevant during clinical laser lithotripsy.

Since bubble dynamics and crater morphology are dominant contributors to retropulsion, two clinical pathways can be envisioned to minimize stone displacement. The first is to use optimized ureteroscope designs that can modulate collapse dynamics and stabilize the standoff distance, enabling efficient energy transfer without excessive retropulsion [39]. The second is to adopt scanning or sweeping techniques rather than drilling at a single point. Because higher crater aspect ratios intensify retropulsion, scanning reduces localized pressure buildup and limits backward stone motion. Conversely, prolonged lasing at one spot amplifies retropulsive forces, which can complicate treatment of impacted stones in confined ureteral regions. Together, these approaches highlight how precise control of fiber positioning and dynamic standoff maintenance can enhance procedural efficiency and safety.

### 2.7 Limitations and Future Directions

The experimental configuration in this study was designed to irradiate the same target location to isolate key parameters influencing retropulsion dynamics. While this approach ensures repeatability, it does not replicate clinical variability, where precise targeting is difficult to maintain. Consequently, the degree of crater‐depth progression achieved experimentally may be overestimated relative to in vivo lithotripsy, leading to higher measured retropulsion velocities. Furthermore, since bubble dynamics were visualized at 40 000 fps, the measured growth and collapse times have uncertainties of approximately ±7.2 and ±10.2μs, respectively. Lighting constraints also limited tracking of individual ejecta particles, and while ejecta‐front velocities were reliably determined, future experiments employing high‐intensity front illumination could enable particle image velocimetry (PIV) for higher fidelity. Ejecta momentum could not be measured directly; instead, ejecta‐front velocity combined with OCT‐derived crater volume was used as a proxy. Additionally, because this study isolates single cavitation events per pulse, multi‐bubble interactions (as encountered in MOSES or pulse‐modulated lithotripters) fall outside its defined scope. While single‐pulse conditions preclude thermal accumulation, continuous clinical operation introduces localized heating, which may alter bubble dynamics and warrants further exploration.

The multivariate scaling analysis developed here provides a descriptive framework rather than a prescriptive model. Begostone samples of uniform size were used for both pulse types, ensuring experimental control but limiting generalization across variable stone compositions. Initial and boundary conditions–such as crater geometry, ambient water temperature, dissolved‐gas concentration, pulse energy, and duration–can influence both ejecta generation and bubble–stone coupling and interaction. Future investigations should systematically vary these conditions, as well as laser modality, pulse energy, and repetition rate, to refine predictive relationships. Additional work could incorporate compressibility‐induced variations in collapse time and vapor‐content‐dependent jet‐impulse attenuation [40]. Such effects, as modeled by compressible‐phase‐transition theories, suggest that vapor content and interfacial heat exchange influence collapse time and jet impulse, leading to deviations from the ideal Rayleigh‐collapse scaling relationship observed in this study. Bubble migration models [41] were excluded because crater confinement and curvature constrain translation, while supercritical vapor compression effects [42] were omitted as they are negligible under our experimental conditions. By quantifying single‐bubble dynamics produced by a clinical Ho:YAG system, this study establishes a transferable framework for interpreting retropulsion mechanisms in future work on pulse‐modulated Ho:YAG and TFL platforms. Moreover, additional dimensionless numbers should be introduced to extend scaling laws to different energy levels and fiber diameters, expanding applicability across lithotripsy systems. In summary, while the present evidence strongly supports that bubble dynamics dominate over ejecta recoil, further validation with broader experimental datasets may be required before drawing definitive conclusions.

## 3 Summary

In summary, this study indicates that stone retropulsion in laser lithotripsy is chiefly governed by vapor‐bubble collapse, rather than by recoil from laser‐induced ejecta. High‐speed imaging and dimensionless modeling show that ejecta act primarily as modulators of retropulsion–shaping bubble dynamics and crater geometry, which subsequently influence momentum transfer to the stone. Short pulses (SPs) rely on impulsive bubble collapse and exhibit strong sensitivity to fiber–stone positioning, whereas long pulses (LPs) benefit from progressive crater deepening and enhanced jet–stone coupling over multiple pulses. A multivariable model captures 86–98% of experimental trends, indicating that crater geometry plays a leading role in LP retropulsion, while inertial scaling and standoff dependence govern SP behavior. Future work will incorporate expanded datasets and cross‐validation to assess predictive power. This ejecta‐modulated cavitation framework clarifies the physical basis of stone motion and provides guidance for optimizing pulse parameters, fiber design, and clinical protocols in lithotripsy and other laser–fluid applications.

## 4 Experimental Section

A Ho:YAG laser (Dornier Medilas H Solvo35, λ=2.1μm) with pulse energy of 0.8J was used in two operational modes (see Figure S14a, and Ho et al. [43]): fragmenting, also called short pulse (SP), and advanced, also known as long pulse (LP). The selected pulse durations effectively represent the clinically and physically meaningful bounds of pulse‐dependent bubble dynamics [44, 45].

In a free field, both modes produce pear‐shaped elongated vapor bubbles [2, 43], with growth times $t_{g}\approx 300\;\mu s$ (for SP) and $t_{g}\approx 325\;\mu s$ (for LP), and collapse times $t_{c}\approx 300\;\mu s$ (for SP) and $t_{c}\approx 250\;\mu s$ (for LP). See Figure S14 for the bubble dynamics in free field. Spherical, soft Begostones [23] of $R_{st}$= 3 mm were glued to the end of a helical spring, with the opposite end held rigidly. The spring's time constant of 96.8 ms ensured that its stiffness did not influence stone dynamics over our observation time (first 2ms, see Section S1.2). A Dornier SingleFlex 400 fiber with a core diameter of 365 μm, and NA = 0.26 was positioned perpendicular to the stone surface, and the fiber‐to‐stone standoff distance (SD) was adjusted using a linear positioning stage. The spring's restoring force ensured that the laser impacted the same location on the stone surface before each subsequent pulse. All experiments were conducted in degassed water at room temperature (25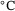). A high‐speed camera (Phantom v7.3; Vision Research, Wayne, NJ) operating at 40 000 fps was used to track the stone motion. This introduces a ±7.2 and ±10.2 μs uncertainty in measuring bubble growth and collapse times, corresponding to ≤ 5 % relative error for SDs > 0.25 mm. For SD = 0 mm, this value reaches a maximum of 16.5% in the growth phase (details in Section S7). Displacement data were extracted using a custom MATLAB script that tracked the center of the stone. Bubble width was obtained by segmenting and isolating the disturbed region above the stone in each frame. The bubble radius was taken to be 0.5$\times \;W_{b,max}$ (Figure 1A). Ejecta dynamics were captured at 5 Mfps using a Kirana 5M camera (Specialized Imaging, UK), synchronized with the Phantom camera, which viewed the setup from a different angle. To avoid energy loss due to contamination in air‐based tests, the fiber was self‐cleaned between each pulse by immersing the contaminated fiber in water and firing five or more pulses. This was found sufficient to restore the energy output from the fiber (measured using Vega ROHS Energy Meter, Ophir Optronics Solutions). Preliminary spatial beam‐profile measurements confirming near‐Gaussian output for both pulse modes are provided in the Section S2. Backlight illumination was provided by a Si‐Lux 640 (Specialised Imaging) for the Kirana camera and a 10 W LED for the Phantom camera. The images used for stone tracking had their resolution enhanced to 3 μm/pixel using Lanczos‐3 image interpolation. Crater morphology was characterized using optical coherence tomography (OCT). Volumes were computed from the cross‐sectional area of the crater, with a resolution of 15μm/pixel in depth and width, and 4μm/pixel in the thickness direction. To avoid error due to repositioning between pulse numbers (PNs), a different set of stones was treated (see Section S1.4) and used for OCT imaging [43].

### 4.1 Statistical Analysis

Statistical analyses were performed to quantify variability and assess the significance of differences in stone retropulsion velocity, bubble dynamics (radius, width, and aspect ratio), crater geometry (depth and profile diameter), and ejecta‐related speed measurements across pulse modes, pulse numbers, and stand‐off distances. Data pre‐processing involved visual inspection of all trajectories and measurement outputs to confirm physical consistency and to exclude spurious tracking artifacts arising from image occlusion by bubbles or ejecta. No data transformation procedures were applied. Outliers were not excluded unless attributable to identifiable experimental or tracking errors. All quantitative results are reported as mean ± standard deviation (SD). Sample sizes (n) are specified for each experiment and correspond to independent stone trajectories or repeated measurements under identical experimental conditions, as indicated in the text, tables, and figure legends. Typical sample sizes ranged from n = 4 to n = 5 per condition. Statistical comparisons between groups were conducted using Student's t‐tests. Paired, two‐side tests were used, with a significance level of p = 0.05. Exact P values are reported where applicable and summarized in Tables S3 and S4. No post‐hoc multiple‐comparison corrections were applied, as comparisons were predefined and limited in number. Regression analyses for scaling relationships and multivariable modeling were performed using least‐squares fitting, and model adequacy was assessed using coefficients of determination ($R^{2}$) and residual analysis to confirm homoscedasticity and absence of systematic bias. All statistical analyses and data processing were performed using MATLAB (MathWorks, Natick, MA).

## Conflicts of Interest

The authors declare no conflicts of interest.

## Supporting information


**Supporting File 1**: advs73819‐sup‐0001‐SuppMat.pdf.

**Supporting File 2**: advs73819‐sup‐0002‐MovieS1.avi.

**Supporting File 3**: advs73819‐sup‐0003‐MovieS2.avi.

**Supporting File 4**: advs73819‐sup‐0004‐MovieS3.avi.

**Supporting File 5**: advs73819‐sup‐0005‐MovieS4.mp4.

**Supporting File 6**: advs73819‐sup‐0006‐MovieS5.mp4.

## Data Availability

The data that support the findings of this study are available from the corresponding author upon reasonable request.
