# Supplementary material for: Ejecta‐Modulated Bubble Dynamics Play a Dominant Role in Stone Retropulsion
Source: Adv Sci (Weinh). 2026 Feb 20;13(22):e16280. doi: 10.1002/advs.202516280 (PMC13088354; doi:10.1002/advs.202516280)
Supplement: Supplementary file 1 — Supporting File 1: advs73819‐sup‐0001‐SuppMat.pdf. [file ADVS-13-e16280-s002.pdf]

Supplementary Information for "Ejecta-Modulated Bubble Dynamics Play a Dominant Role in Stone Retropulsion"

Obed S. Isaac[†*] *Arpit Mishra*[†*] *Georgy N. Sankin* *Junqin Chen* *Pei Zhong*[*]

O. S. Isaac, A. Mishra, J. Chen
Thomas Lord Department of Mechanical Engineering and Materials Science, Duke University, Durham, NC 27708, United States
G. N. Sankin
Department of Biomedical Engineering, Tulane University, New Orleans, LA 70118, United States
P. Zhong
Thomas Lord Department of Mechanical Engineering and Materials Science, Duke University, Durham, NC 27708, United States
Email Address: pzhong@duke.edu

SI1. Experimental Set-Up

All experiments were conducted using a custom-built setup designed to characterize stone retropulsion dynamics induced by laser-driven bubble activity (Figure 1). A Holmium:YAG laser system (Dornier Medilas H Solvo 35, $\lambda = 2.1$ μm) operating at a pulse energy of 0.8 J was coupled to a 400 μm core optical fiber (Dornier SingleFlex 400, NA = 0.26). The fragmenting mode (short pulse, FWHM: 80μs) and advanced mode (long pulse, FWHM: 205μs) were used for these experiments. These durations were measured from their temporal pulse power profiles in air using an InGaAs photodetector (PDA10D; Thorlabs, Newton, NJ) [5]. The profiles are shown in Figure 14A), free-field bubble images (Figure 14B), and their height and width have been given in Figures 14A &D. The knife-edge profiles for the laser pulse on output plane can be expected to follow the same Gaussian spatial characteristics as that of Lee et al. [6], given that our laser system uses comparable fiber geometry (400 μm core, NA $\approx$ 0.26) and operates under similar pulse energies (0.8 J). They demonstrated that undamaged 272–365 μm Ho:YAG fibers produce a near-Gaussian spatial beam profile at the distal tip, with a full-width at half-maximum (FWHM) of approximately 350 μm, which closely matches the fiber core diameter. Their findings also show that bending or moderate fiber wear only slightly flattens this Gaussian shape without significantly altering the spatial symmetry or energy distribution. The difference between SP (80 μs) and LP (205 μs) settings lie entirely in the temporal domain, not in the beam geometry. This interpretation is supported by Fried et al. [4], who reviewed the optical characteristics of Ho: YAG and Thulium-based lithotripsy lasers and confirmed that the Holmium laser beam delivered through standard silica fibers is multimode, but it approximates a Gaussian output profile due to the strong absorption and thermal diffusion at 2120 nm. The fiber was oriented perpendicular to the stone surface, and its axial position relative to the stone was precisely controlled. Stand-off distances (SDs) between the fiber tip and stone surface were varied systematically, and positioning repeatability was verified before each run.

SI1.1 Tracking Stone Motion:

To measure stone displacement, soft artificial kidney stones were mounted on a spring-loaded platform (time constant 96.8 ms), allowing for free axial motion in response to laser pulses. The position of the stone was tracked in real time using high-speed video from Phantom v7.3 (40,000 fps) or Kirana (5,000,000 fps), depending on the temporal resolution required. Stone trajectories were extracted using a custom centroid-tracking MATLAB algorithm for the stone. The images were enlarged tenfold using Lanczos-3 interpola-

[*]Corresponding authors
[†]These authors contributed equally to this work.

tion implemented in MATLAB. This gives sub-pixel accuracies that are correct up to $1/10^{th}$ of the original pixel size. The calibration factors of our raw images decreased from roughly 30 μm to approximately 3 μm. Since the actual stone displacement is very small, stone retropulsion is not very obvious in these images (Figures 1 and 2). A useful method to visualize stone movement is provided in Figure 2 for SD = 0.5 mm, in terms of a composite streak image derived from the individual frames. Stone displacement was not tracked using the streak image as we found the stone boundary detection to be highly sensitive to the choice of edge detection in MATLAB. The centroid tracking was a more robust method as it is not sensitive to variations in the intensity of light and the presence of bubble/ejecta particles on top of the stone. Retropulsion velocities were obtained from the average slopes of the steady region (Figure 1D) for n=4 or n=5 trajectories. The data and their p-values have been tabulated in Tables 2, 3, & 4. Group differences were assessed with two-sample Welch's t-tests (unequal variances) using MATLAB's ttest2 function. p-values were computed from the t-distribution with degrees of freedom estimated via the Welch–Satterthwaite approximation.

The growth time (t_g) and the collapse time (t_c) of the bubbles were obtained from the Phantom camera images at F=40,000 fps.

Craters were measured after the treatment using optical coherence tomography (OCT) (OQ Labscope, Lumedica, Durham, NC). A script written using MATLAB (MathWorks, Natwick, MA) was used to extract crater information such as the volume (V_c), maximum depth (d_c), and area of the surface profile (A_c). The resolution was 15 μm/pixel in depth and width, and 4 μm/pixel along the breadth. Comparative volume information is provided for stones in water and in air for LP and SP at SD=0.5 mm (Table 1).

Bubble and ejecta dynamics were visualized using LED shadowgraphy, with timing synchronized to the laser pulse using an external trigger. Ejecta velocity was quantified by frame-by-frame analysis, where pixel intensity streaks were analyzed to obtain the front-edge trajectory of the ejecta. Bubble dynamics were characterized over a 25 μs resolution timescale, capturing growth and collapse phases. All experiments were performed in a room-temperature-controlled environment using degassed water to minimize the effect of dissolved gases on cavitation dynamics. The laser energy output was monitored prior to each trial and was maintained within $\pm 2\%$ of the target pulse energy.

SI1.2 Justification for Neglecting Spring Reaction Force in Stone Dynamics

The spring-loaded platform used to mount the stone had a time constant of approximately 96.8 ms (Figure 3). This value represents the characteristic timescale over which the spring–mass system responds to an external disturbance. In contrast, the physical processes that induce stone retropulsion–namely laser pulse emission, ejecta expulsion, and cavitation bubble dynamics (growth and collapse)–occur over much shorter timescales, typically between 0.1 and 1 ms [1, 13]. Because these processes unfold nearly two orders of magnitude faster than the spring's characteristic response, the restoring force from the spring remains effectively dormant during the initial stone motion.

This assumption is supported by classical transient vibration theory. For a spring–mass system subjected to an impulsive or short-duration force, the force duration is too brief for the spring to respond dynamically. In such cases, the mass accelerates nearly freely, and the spring force remains non-impulsive, exerting negligible influence on the immediate momentum change [10]. The spring only influences the system response in subsequent oscillations after the impulse has ceased.

From the time constant (τ_s) of the spring (Fig. 3), the stiffness(k_{sp}) of the spring was estimated to be 1.374 N/m using the equation below, where $m_{total} = m_{st} + m_a$.

$$\tau_s = 2\pi\sqrt{(}m_{total}/k_{sp})$$

For SD = 1 mm, where the stone displacement Δ_{max} is the highest (see §2.2.3), the spring force value, $F_{sp} = -k_{sp} \times \Delta_{max} = 0.206\ mN$. We use this case to demonstrate that the spring force is negligible compared to that from the bubble. An approximation for the internal pressure inside the bubble was

obtained by solving the Rayleigh-Plesset equation. The initial pressure and starting radius of the bubble were taken as 5 bar at 1 mm to match the experimentally observed value: maximal bubble radius, R_{max} of 2.19 mm and the duration of the first bubble cycle $(t_c + t_g) = 0.6\ ms$. The ambient pressure had to be set as 80 kPa, to ensure that output of the Rayleigh-Plesset calculations matched the experimental data. The evolution of the bubble radius is shown in Fig. 4A. Then, the contact area of the bubble was obtained assuming that it is proportional to the evolving bubble size (i.e., the experimental R vs t plot). Using this, the bubble force F_b on the spring was estimated by neglecting drag and the surrounding ambient pressure. The spring force F_{sp} was estimated as $-k_{sp}\Delta x_{st}$, using stone displacement data Δx_{st}, extracted from the high speed images (Figure 1A). It needs to be mentioned that these values as conservative as we have assumed that the spring responds instantly to the displacement despite having a $\tau_{sp}/(t_g + t_c) \gg 1$. The evolution of the bubble and spring force values are shown in Figure 4B for the first bubble cycle, and their ratio is shown in Figure 4C.
Figure 4C confirms that within the relevant impulse time scale of cavitation-induced retropulsion (Table 5), the spring has insufficient time to generate an appreciable restoring force. Therefore, during the early phase of motion, the system behaves in a near-inertial regime.
The restoring spring force contributes <0.5% of the total force during the initial cycle where the forces are the highest for SD = 1 mm (Fig. 4, Table S5). Thus, the initial retropulsion dynamics occur in a quasi-free inertial regime dominated by bubble-driven forces directly reflecting the hydrodynamic impulse from the bubble and ejecta dynamics and not the influence of spring support mechanics.

SI1.3 Stone Preparation Protocol: Soft BegoStone™ Fabrication and Properties

Artificial soft kidney stones were fabricated using BegoStone^{TM} (Bego, Bremen, Germany), a plaster-based material commonly used in dental applications and adapted for spherical stone preparation [3]. The powder composition primarily includes calcium sulfate hemihydrate (85–90%), along with accelerator/retarder additives (5–10%) and pigments or stabilizers (1–5%). For soft stone fabrication, a powder-to-water ratio of 5:2 by weight was used. The BegoStone^{TM} powder was measured using an analytical balance in a vibration-free environment, and water was added incrementally using a syringe with a needle for precision. After thorough mixing for approximately 30–60 seconds to ensure a uniform slurry, the mixture was poured into cleaned and dried spherical molds (typically 6 mm in diameter). Care was taken to stir the slurry between each mold filling to avoid settling and preserve homogeneity. Mold inserts were added post-filling when required to control geometry. Curing was performed at room temperature (23 ± 2°C) for at least 24 hours in a humidity-controlled environment. Prior to water-based experiments, stones were soaked in degassed water for at least 20 minutes to ensure hydration equilibrium. The typical mass of soft spherical stones ranged from 190 ± 20 mg.

SI1.4 Experiments in Water

For underwater experiments, stones were immersed in degassed water for at least 20 minutes prior to laser pulse application. In multiple-pulse experiments, precise fiber positioning relative to the stone's surface was required to maintain a consistent stand-off distance (SD) as removing stones for optical coherence tomography (OCT) measurements disrupted this positioning. Therefore, to avoid repositioning errors during OCT, a separate stone set was used post-experiment. Three repeats were used for OCT measurement. At least 4 stones were used for the other experiments.
For stand-off distances $(SD) \geq R_{max} \approx 3$ mm, where the laser-induced bubble does not contact the stone, all pulses (PNs 1–5) are considered repeated trials under the same conditions.

SI1.5 Experiments in Air

Wet stones were prepared by soaking dry stones in degassed water for at least 20 minutes, then gently blotted with absorbent paper to remove excess surface water. Between experimental runs, the stone was removed, and the fiber was cleaned using multiple laser firings underwater to remove potential ejecta residue. Laser energy remained consistent across experiments and being measured using Vega ROHS Energy Meter, Ophir Optronics Solutions. At least 4 replicates were used for mean and standard deviation calculations. For single-pulse experiments conducted in air, the resulting craters on the stones were analyzed using optical coherence tomography (OCT).

SI2 Spatial Beam-Profile Characterization of the Ho:YAG Laser

Preliminary spatial beam-profile measurements were performed to assess the optical output characteristics of the Ho:YAG laser used in this study. Measurements were conducted using a pyroelectric camera (Pyrocam IV, SP90404, MKS–Ophir Systems) on a freshly cleaved optical fiber (Dornier SingleFlex 400 fiber, core diameter: 365 μm, NA = 0.26) operated in short-pulse (SP) and long-pulse (LP) modes. The bare fiber output was directed onto a dual-wedge beam splitter (LBS-300s-UV) to attenuate the beam prior to imaging on the camera sensor. Beam profiles were obtained by averaging 10 pulses in the camera's software, with the laser firing 200 pulses at 10 Hz, and spatial intensity distributions were extracted along two orthogonal axes. Representative beam-profile images and corresponding Gaussian fits are shown in Figure 15.

Both SP and LP modes exhibited near-Gaussian spatial intensity distributions. The goodness-of-fit values for the Gaussian models were 0.887 for SP and 0.908 for LP, consistent with the expected deviation introduced by the noisy tail, the sources of which are discussed in the following paragraph. A modest anisotropy was observed for the LP mode, which exhibited an approximately 15% larger beam width along one axis, while remaining comparable to the SP mode along the orthogonal direction.

Absolute spatial beam widths at the fiber tip are not reported here. Due to the absence of a wavelength- and power-matched collimator and the use of a dual-wedge beam splitter, the diverging beam expanded across the thickness of the splitter and approached the size of the detector active area. This introduced elevated background noise and limited the ability to reliably calibrate the distance between the fiber tip and the effective detector plane.

Nevertheless, the measured spatial distributions directly demonstrate that both pulse modes produce near-Gaussian beam profiles with broadly similar spatial geometry, consistent with prior pyroelectric-camera measurements of Ho:YAG fiber outputs reported in the literature [6]. Future measurements using optimized attenuation and collimation will enable fully calibrated beam-width characterization.

SI3 Ejecta Speed Measurement

From high-speed images (5 Mfps), the ejecta front was difficult to locate precisely (zoomed region between fiber and stone in Figure 5). We extracted the highest pixel intensities across rows in a zoomed region to form "streak images" (Figure 5). The progression of the ejecta front from the appended synthetic images is clearly visible, and the slope was used to obtain the ejecta speed, v_{ej}. Although the ejecta amount reduces with PN, the average speed changes minimally. This method was also applied underwater (Figure 6).

To understand if the particles can influence the bubble morphology, two possible mechanisms - one mechanical, and the other thermal, are explored in the next section.

SI3.1 Particle Response and Stokes Number Analysis

To consider whether the solid fragments (ejecta) in our experiments follow the flow (i.e., have a tracer-like behavior) or if they are able to perturb the bubble interface, the viscous response time relative to

the characteristic collapse time needs to be evaluated. For this analysis, the particle response (Stokes) time is given by

$$\tau_p = \frac{\rho_p d_p^2}{18\mu}, \qquad St = \frac{\tau_p}{t_{char}},$$

where ρ_p is the particle density, d_p is the particle diameter, μ is the dynamic viscosity, and t_{char} is the bubble collapse time. For $St \ll 1$, particles follow the flow as tracers; for $St \gtrsim 1$, they lag and can modify the local hydrodynamics.

Representative Experimental Parameters

$$\mu = 1 \times 10^{-3} \text{ Pa} \cdot \text{s},$$
$$\rho_p \approx 2000 \text{ kg/m}^3,$$
$$t_{char} \sim 0.6 \text{ ms} \quad \text{(Rayleigh collapse time for } R_{\max} \sim 2.5 \text{ mm).}$$

Particle Motion in Water

- $d_p = 200\ \mu\text{m}: \quad \tau_p \approx 4.44 \times 10^{-3} \text{ s} = 4.4 \text{ ms}, \quad St \approx 7.5\ (\gg 1).$
  Such large fragments will not act as tracers and can substantially perturb the bubble interface.
- $d_p = 20\ \mu\text{m}: \quad \tau_p \approx 4.44 \times 10^{-5} \text{ s} = 0.075 \text{ ms}, \quad St \approx 0.075.$
  Marginal behavior: these particles partly follow the flow but may lag during the fastest collapse transients, as seen in supplementary Movies #4 and #5.
- $d_p = 5\ \mu\text{m}: \quad \tau_p \approx 2.78 \times 10^{-6} \text{ s} = 2.78\ \mu\text{s}, \quad St \ll 0.005.$
  These are good tracers.

Particle Motion in Air or Vapor For air or representative steam/vapor ($T \approx 373$ K):

$$\mu_{\text{air}} = 1.8 \times 10^{-5} \text{ Pa} \cdot \text{s}, \qquad \mu_{\text{steam}} = 1.23 \times 10^{-5} \text{ Pa} \cdot \text{s}.$$

- $d_p = 200\ \mu\text{m}: \quad \tau_p \approx 0.25 \text{ s} = 250 \text{ ms}, \quad St \approx 411\ (\gg 1).$
  Ballistic; fully decoupled from the flow.
- $d_p = 20\ \mu\text{m}: \quad \tau_p \approx 2.5 \times 10^{-3} \text{ s} = 2.5 \text{ ms}, \quad St \approx 4.1.$
  Largely ballistic and decoupled.
- $d_p = 5\ \mu\text{m}: \quad \tau_p \approx 1.54 \times 10^{-4} \text{ s} = 154\ \mu\text{s}, \quad St \ll 1.$
  Acts as a marginal tracer in air.

Interpretation From this scaling, particles smaller than roughly $40-70\ \mu$m ($St \approx 1$) act as passive or marginal tracers for the collapse times measured in our system, whereas larger fragments move ballistically and can perturb the bubble interface.
In air, $20-200\ \mu$m fragments have very large Stokes numbers ($St = 4-400$), implying ballistic behavior. It is to be noted that owing to the turbulent nature of the flow inside the bubble, a better characteristic time t_{char} would be to use the Kolmogrov time scale. This is of the order of a microsecond, resulting in the particles acting like ballistic particles inside the vapor-filled bubble. These ejecta move quasi-independently of the gas flow and can interact mechanically and thermally with the expanding vapor bubble. Upon entering the liquid phase, viscous drag rapidly damps their motion, after which they behave as tracers of the bulk flow.
Previous lithotripsy studies report that most ejecta generated in "dusting" regimes are fine ($< 200\ \mu$m, often $\ll 100\ \mu$m). Our high-speed images confirm that the dominant ejecta population (from our videos, see Videos #1, #4, #5) lie well below this scale, consistent with tracer-like behavior for most particles.

For momentum analysis, we therefore measured only the ejecta-front velocity (the leading edge) and combined it with OCT-derived crater data and a conservative size distribution to estimate an upper bound on total ejecta impulse.

SI3.2 Thermal Coupling

When solid fragments traverse the vapor–liquid interface ballistically, they can scatter and locally deposit laser-absorbed heat at the bubble boundary. Following the mechanism described by Zhao *et al.* [16], such localized radiative heating can momentarily enhance surface vaporization, producing lobe-like elongations along the laser axis (as also seen in our supplementary frames). Once these particles re-enter the liquid medium, their velocities quickly decay due to viscous drag, and they subsequently move with the surrounding flow, behaving as tracers of the bulk bubble-induced motion.
Thus, while large fragments ($> 70\ \mu$m) may transiently perturb the interface through thermal and radiative coupling, most particles in our regime are fine enough to serve as effective tracers after losing momentum in the bulk liquid. This two-stage behavior—ballistic scattering followed by hydrodynamic entrainment—is consistent with the laser-mediated advection–phase-transition interaction outlined by Zhao *et al.* (2024) for elongated, lobe-forming bubbles.

SI4 Jet-Velocity Scaling under Evolving Boundary Conditions

SI4.1 Curvature correction factor to R_{max}

From the work of Tomita et al. [14] we obtained a corrected version of R_{max}, viz., R^*_{max}, by solving the following equation

$$\frac{1}{KR^*_{\text{max}}}\left(\frac{\tau_c v_{\text{ref}}}{k_1 R^*_{\text{max}}}-1\right)=\frac{R_{st}}{SD_e(2R_{st}+SD_e)}-\frac{1}{R_{st}}\ln\left(\frac{(R_{st}+SD_e)^2}{SD_e(2R_{st}+SD_e)}\right).$$

Here, K=0.4065, k_1=0.914, $v_{ref} = \sqrt{(}p_\infty/\rho_w)$. Where, we substitute $p_\infty = 1.013 \times 10^5$ Pa and $\rho_w = 1000\ kg/m^3$ to yield $v_{ref} = 10.07\ m/s$. The curvature factor is incorporated using the stone radius, R_{st}= 3 mm. t_c is the collapse time obtained from the experiment.

SI4.2 Influence of Correction Factors

The original work by Outi *et al.* [11] on jet-velocity formulation assumes a single anisotropy parameter based on the Kelvin impulse, which yields a

$$v_{\text{jet}} \propto \gamma^{-2}$$

scaling for bubbles near stationary rigid walls. This original model cannot fully capture the complex conditions present in our experiments. For our configuration, the stone boundary is curved, cratered, and dynamically evolving, such that the effective stand-off distance (SD_e) changes with successive pulses as the crater depth increases.
This discrepancy is now captured in Figure 16, which compares the measured normalized retropulsion velocity to three progressively refined estimates of the collapse-jet speed used for normalization. Panel A shows the result obtained when using the classical Outi *et al.* [11] jet-velocity scaling (using the original stand-off SD), which predicts the well-known power-law $v_{\text{jet}} \propto \gamma^{-2}$. The data do not collapse under this normalization: substantial scatter and systematic offsets remain because the original formulation assumes a single, stationary planar wall and does not account for the evolving crater geometry or finite curvature of the stone surface.

To address this limitation, we extended Outi's model by replacing the nominal stand-off parameter (SD) with a pulse- and time-dependent effective parameter defined as

$$SD_e = SD + d_c,$$

which accounts for the evolution of crater depth (d_c) with each laser pulse. Defining the effective stand-off distance parameter $\gamma_e = SD_e/R_{\text{max}}$, Panel B shows the normalization v_r/v_{jet} $vs.$ γ_e using the Outi functional form with the substitution $\gamma \mapsto \gamma_e$. This modification markedly reduces scatter: data from different pulse numbers and pulse durations fall onto a much tighter locus, demonstrating that crater evolution is a first-order modifier of jet production. This adjustment directly incorporates the experimentally measured crater geometry obtained from OCT data and therefore better represents the instantaneous confinement experienced by the bubble during collapse.
Furthermore, to include the influence of surface curvature and finite stone-size effects, the correction from Tomita *et al.* [14] for bubble dynamics near curved rigid surfaces was incorporated into the modified formulation based on the SD_e parameter. In Tomita's framework, the curvature parameter ξ modifies the prolongation coefficient μ, and hence the bubble-collapse time and jet velocity. Convex geometries ($\xi < 1$) yield shorter collapse times compared to Rayleigh collapse times (see Fig. 12a). By introducing this curvature-dependent correction into the modified Outi scaling, the jet-velocity relation effectively couples wall curvature (stone radius R_{st}, for spherical surfaces), crater depth (d_c), and the evolving effective stand-off distance (SD_e), thereby capturing more realistic boundary conditions representative of our experimental system, as shown in Panel C.
The result (Panel C) yields the tightest collapse: low-γ_e points (strong confinement / small effective stand-off) and data across pulse modes now align with the same trend and reduced residuals. This improvement is particularly significant for cases with pronounced curvature (small local radius of curvature) where Tomita predicts reduced prolongation ($\mu < 1$) and therefore faster collapse and higher jet speeds than planar-wall theories would suggest. This agreement further confirms that the Outi [11] and Tomita [14] corrected scaling, accounting for both crater geometry and surface curvature, provides a quantitatively robust and physically grounded normalization.

SI5. Relevance of Other Forces

In this section, we quantitatively evaluate other physical mechanisms that could, in principle, contribute to stone retropulsion, and assess their relative magnitudes compared with experimentally observed retropulsion velocities.

SI5.1 Radiation Pressure

Radiation pressure arises from photon momentum transfer and could, in principle, contribute under contact-mode conditions (SD = 0 mm). To estimate the radiation pressure p_{rad}, we use the conservative upper bound corresponding to a fully reflecting surface,

$$p_{\text{rad}} = \frac{2I}{c},$$

where I is the laser intensity and c is the speed of light. For a pulse energy $E_p = 0.8$ J and full-width-at-half-maximum (FWHM) pulse durations of 80 μs and 205 μs for short-pulse (SP) and long-pulse (LP) modes, respectively, the peak power is estimated as E_p/t_{FWHM}. The spot size was conservatively taken to be equal to the fiber core diameter (365 μm).
Using these parameters, the resulting radiation pressures are **642 Pa** for SP and **250 Pa** for LP. These values are several orders of magnitude smaller than the pressures required to produce the observed stone retropulsion velocities and are therefore negligible contributors to stone motion.

SI5.2 Ablation and Recoil Pressure

The combined contribution of ablation pressure and recoil pressure from ejecta momentum flux was estimated using Equation A11 from the work of Cummings and Walsh on Er:YAG laser-induced tissue tearing [2]:

$$p_{\mathrm{abl}} + \frac{m_{\mathrm{ej}}\,\Delta v}{A\,\Delta t} = A\exp\left(-\frac{U}{RT}\right) = \frac{I}{L}(RT)^{1/2}\left(\sqrt{\gamma} + \frac{1}{\sqrt{\gamma}}\right).$$

Here,

- I: Ablation energy flux, and not irradiance as mentioned in the original paper. This is calculated as $\rho_{solid} \times v_{ablation} \times L$, where ρ_{solid} is the density of the solid medium, L is the latent heat of vaporization, and $v_{ablation}$ is the speed of the ablation front in the solid (see Equation A4 [2])
- p_{abl}: pressure at the ablated surface,
- m_{ej}: mass of ablated material ejected during the interval Δt,
- Δv: ejecta velocity (v_{ej} in the present experiments),
- $A = 3.075 \times 10^{10}$ Pa: Arrhenius prefactor for saturated vapor pressure of water,
- Δt: characteristic ejection timescale,
- U: activation energy for vaporization, with $U/R = 4688.2$ K,
- R: gas constant for steam (=461.5 $\mathrm{J\,kg^{-1}K^{-1}}$),
- T: absolute temperature at the ablation surface,
- $L = 4.2 \times 10^6$ $\mathrm{J\,kg^{-1}}$: effective latent heat of vaporization,
- $\gamma = C_p/C_v = 4/3$, ratio of specific heats.

The left-hand side represents the total surface pressure, composed of the ablation pressure p_{abl} and the recoil pressure due to ejecta momentum flux. The right-hand side represents the saturated vapor pressure, expressed in Arrhenius form.

Recoil Pressure: To estimate the recoil pressure, OCT-derived crater geometry from PN1 was used to calculate the ablated mass and surface area. v_{ej} measured at SD = 0.5 mm were used for Δv. This yields recoil pressures of **145 Pa for SP** and **62.9 Pa for LP**.

Ablation Pressure: For the pressure term on the right, $I = \rho_{solid} \times v_{ablation} \times L$, where ρ_{solid} is the density of Begostone = 1700 kg/m^3, L is the latent heat of vaporization. The speed of the ablation front is not available in the literature. However, we can estimate the upper limit from the depth of the crater after PN1, and divide it by the pulse duration. This would yield $v_{ablation}$ of 1 m/s for SP and 0.7 m/s for LP. In the absence of a better estimate, for L, we use 4.2×10^6 $\mathrm{J\,kg^{-1}}$ as used in Cummings and Walsh [2] for their work on tissue ablation for Er:YAG lasers. This yields extremely high ablation pressures p_{abl} of 44 and 17 GPa for SP and LP. This number is quite obviously an overestimate (5.5 and 2.2 kN) as the forces from this event are known to be of the order of a few newtons only [8]. Nevertheless, this proves to show that the ablation pressures is one of the dominant mechanisms for retropulsion in air. However, in water, there are several attenuation mechanisms in play.

- Water absorbs 98% [15] of the laser energy.
- And second, from our high-speed videos (see Movies 2 to 5), ejecta emerge in spurts. This implies that the laser interaction with the stone is not continuous, and the average ablation speeds are expected to be lower.

- Third, the delay due to inertia of the stone and added mass would mean that this force would result in visible stone motion only during the suction phase of the bubble as discussed in Sec 2.5. And then, since the stone motion has been shown to be in synchrony with bubble motion (Sec 2.1), it demonstrates the dominating role of the bubble forces for underwater interactions.

CFD simulations planned for future work will enable extraction of the exact bubble-induced forces, allowing quantitative comparison and highlighting the larger effective force area associated with bubble–stone interactions.

SI5.3 Vapor Bubble Collapse Pressure

To directly measure pressure transients associated with cavitation, an ONDA HNC-1000 needle hydrophone was positioned approximately 10 mm from the fiber tip. Measured pressure signals were scaled to an equivalent distance of 1 mm using spherical spreading ($1/r$) and temporally corrected using a sound speed of 1480 m/s in water. Experiments were conducted for one ablative condition (SD = 0.25 mm) and two non-ablative conditions (SD = 3 mm and 4 mm). The measured retropulsion velocities (v_r) and peak pressures were tabulated in Table 6.
The data show no meaningful correlation between peak pressure magnitude and stone retropulsion velocity. Although peak pressures (p_{max}) increase modestly with pulse number for SD = 0.25 mm, these increases are not statistically significant. Moreover, for SD = 3 mm and 4 mm, peak collapse pressures are comparable to or higher than those at SD = 0.25 mm (as measured 1 mm from the fiber tip, and away from the stone), yet retropulsion velocities are substantially smaller. The peak pressures occur much before the inflection point (t_{IP}). Pressure levels within a narrow window preceding the inflection point (approximately 100 μs, $P_{IP-100\mu s}$) were also examined to account for inertial delay in stone response (Fig. 17). These pressures were found to be typically in the range of 0.2–0.3 MPa and persist for approximately 10 μs, and occur between the time t_{max} where the p_{max} event occurs (usually) at first collapse, and the inflection point, t_{IP}. This time has also been tabulated as $t_{IP-100\mu s}$ in Table 6. An impulse-based estimate shows that such pressure transients could impart velocities of at most $\sim$ 2 mm/s to a 200 mg stone. Accounting for added mass in water further reduces this estimate to $\sim$ 1.5 mm/s, more than an order of magnitude smaller than the experimentally observed retropulsion velocities ($\sim$ 35 mm/s). Collectively, these results demonstrate that radiation pressure, ablation recoil, and acoustic pressure transients are all insufficient to account for the observed stone motion. Instead, the data strongly supports the conclusion that stone retropulsion is dominated by bulk fluid momentum associated with asymmetric vapor bubble collapse and collapse-jet formation in the immediate vicinity of the stone surface.

SI6. Multivariable Scaling Analysis of Ejecta-Modulated Bubble-Induced Stone Motion

SI6.1 Derivation of Dimensionless Parameters from the Rayleigh–Plesset Equation

Governing Equation The dynamics of a spherical cavitation bubble in an incompressible liquid are described by the Rayleigh–Plesset (RP) equation:

$$\rho\left(R\ddot{R}+\tfrac{3}{2}\dot{R}^2\right)=p_b(t)-p_\infty-\frac{2\sigma}{R}-\frac{4\mu\dot{R}}{R},$$

where $R(t)$ is the instantaneous bubble radius, ρ is the liquid density, $p_b(t)$ is the bubble internal pressure, p_∞ is the ambient pressure, σ is the surface tension, and μ is the liquid viscosity.

Under the inertial regime relevant to laser-induced cavitation, viscous and surface-tension terms are negligible, reducing the RP equation to

$$\rho\left(R\ddot{R}+\tfrac{3}{2}\dot{R}^2\right)m \approx p_b(t)-p_\infty.$$

At maximum expansion $R = R_{\max}$ and subsequent collapse, the characteristic driving pressure is

$$\Delta P = p_\infty - p_v,$$

and the corresponding collapse velocity scale is obtained from an energy balance:

$$v_{\text{ref}} \sim \sqrt{\frac{\Delta P}{\rho}}.$$

Dimensionless Analysis

The stone retropulsion velocity v_r is driven by the momentum transfer from the collapsing asymmetric bubble. The quantities influencing this process are:

$$v_r = f(R_{\max}, SD_e, t_c, t_g, D_c, d_c, A_c).$$

By dimensional analysis (Buckingham Π theorem) with nine variables and three fundamental dimensions (M, L, T), we obtain six dimensionless groups:

$$\Pi_1 = \frac{v_r}{v_{\text{ref}}},\quad \Pi_2 = \frac{SD_e}{R_{\max}},\quad \Pi_3 = \frac{t_c}{t_c+t_g},\quad \Pi_4 = 1+\frac{R_{\max}}{D_c},\quad \Pi_5 = 1+\frac{d_c}{D_c},\quad \Pi_6 = 1+\frac{A_c}{SD_e^2}.$$

Hence, the general dimensionless form is

$$\frac{v_r}{v_{\text{ref}}} = C\,\Pi_2^{\alpha}\Pi_3^{\beta}\Pi_4^{\eta}\Pi_5^{\kappa}\Pi_6^{\zeta}.$$

SI6.1 Physical Interpretation

Each exponent $(\alpha, \beta, \eta, \kappa, \zeta)$ represents the weighting of a corresponding physical mechanism:

- Π_2: geometric proximity / collapse asymmetry,
- Π_3: energy concentration during collapse,
- Π_4 and Π_5: confinement and jet focusing,
- Π_6: coupling efficiency and effective contact area.

Regression against experimental data determines the empirical coefficients while maintaining theoretical consistency with RP scaling. Thus, the above equation represents a dimensionless reduction of the Rayleigh–Plesset equation under the experimental regime, retaining its inertial scaling ($v_r \propto \sqrt{\Delta P/\rho}$) while incorporating the geometric and temporal parameters that control asymmetric bubble collapse and retropulsion.
The predicted retropulsion velocity $v_{\text{r,p}}$ is modeled as a function of geometric, temporal, and morphological parameters:

$$\frac{v_{\text{r, p}}}{v_{\text{ref}}} = C\left(\frac{SD_e}{R_{\max}}\right)^{\alpha}\left(\frac{t_c}{t_g+t_c}\right)^{\beta}\left(1+\frac{R_{\max}}{D_c}\right)^{\eta}\left(1+\frac{d_c}{D_c}\right)^{\kappa}\left(1+\frac{A_c}{SD_e^2}\right)^{\zeta} \tag{1}$$

Here, C is a dimensionless empirical coefficient obtained by regression, and $v_{\text{ref}} = \sqrt{\frac{p_\infty}{\rho_w}} =$

$\sqrt{101325\ [Pa]/1000\ kg/m^3} \approx 10\,\mathrm{m/s}$ is the reference velocity associated with bubble collapse in water [9]. The variables SD_e, t_c, t_g, $R_{\max}$, d_c, D_c, and A_c represent the stand-off geometry, bubble collapse/growth timing, bubble size, and crater morphology parameters influencing the interaction between the cavitation bubble and the stone. Some of these variables were designed to ensure continuity of the regression model, especially, $1 + \frac{R_{\max}}{D_c}^{\eta}$, to avoid divergence in the regression model for $D_c \to 0$. This additive normalization stabilizes the scaling near contact-mode conditions and facilitates comparison between confined and unconfined regimes. The exponents $\alpha, \beta, \eta, \kappa, \zeta$ were determined via multivariate regression using experimental data across all pulse numbers (PNs) and stand-off distances (SDs), separately for short pulse (SP) and long pulse (LP) modes.
Based on the fitted coefficients, the equations for long-pulse and short-pulse retropulsion velocity are given by:

Long Pulse:

$$\frac{v_{\mathrm{r,p,LP}}}{v_{\mathrm{ref}}} = 1.445 \times 10^{-4} \cdot \left(\frac{SD_e}{R_{\max}}\right)^{2.390} \left(\frac{t_c}{t_g + t_c}\right)^{-1.106} \left(1 + \frac{R_{\max}}{D_c}\right)^{0.564} \left(1 + \frac{d_c}{D_c}\right)^{5.634} \left(1 + \frac{A_c}{SD_e^2}\right)^{1.344}$$

Short Pulse:

$$\frac{v_{\mathrm{r,p,SP}}}{v_{\mathrm{ref}}} = 2.504 \times 10^{-2} \cdot \left(\frac{SD_e}{R_{\max}}\right)^{2.035} \left(\frac{t_c}{t_g + t_c}\right)^{2.219} \left(1 + \frac{R_{\max}}{D_c}\right)^{0.646} \left(1 + \frac{d_c}{D_c}\right)^{-0.070} \left(1 + \frac{A_c}{SD_e^2}\right)^{1.183}$$

For the specific case of a 3 mm radius stone used in the experiments ($R_{\mathrm{stone}} = 3\,\mathrm{mm}$), we can rewrite the expressions by substituting the geometric dependencies explicitly:

Simplified LP Equation (for $R_{\mathrm{st}} = 3\,\mathrm{mm}$)

$$\frac{v_{\mathrm{r,p,LP}}}{v_{\mathrm{ref}}} = \frac{1.445 \times 10^{-4} \cdot \left(1 + \frac{R_{\max}}{D_c}\right)^{0.564} \left(1 + \frac{d_c}{D_c}\right)^{5.634} \left(1 + \frac{\pi}{4} \cdot \frac{D_c^2}{SD_e^2}\right)^{1.344}}{\left(\frac{SD_e}{R_{\max}}\right)^{2.390} \left(\frac{t_c}{t_g + t_c}\right)^{1.106}}$$

Simplified SP Equation (for $R_{\mathrm{st}} = 3\,\mathrm{mm}$)

$$\frac{v_{\mathrm{r,p,SP}}}{v_{\mathrm{ref}}} = \frac{2.504 \times 10^{-2} \cdot \left(\frac{t_c}{t_g + t_c}\right)^{2.219} \left(1 + \frac{d_c}{D_c}\right)^{-0.070} \left(1 + \frac{\pi}{4} \cdot \frac{D_c^2}{SD_e^2}\right)^{1.183}}{\left(\frac{SD_e}{R_{\max}}\right)^{2.035} \left(1 + \frac{R_{\max}}{D_c}\right)^{0.646}}$$

These equations quantitatively capture how retropulsion velocity scales with the evolving geometry of the crater, the bubble dynamics, and the fiber–stone spatial configuration. They also reflect distinct driving mechanisms in the short- and long-pulse regimes: the LP case is more sensitive to crater evolution and coupling efficiency (via κ), while SP is more influenced by the timing of bubble collapse and spatial proximity (via α and β).
The high predictive power of these models is reflected in their coefficient of determination: $R^2 = 0.981$ for long pulse and $R^2 = 0.864$ for short pulse.

SI6.2 Relative Contributions of Dimensionless groups

Consider the proposed descriptive formula for stone retropulsion velocity,

$$v_r = A \prod_{j=1}^{5} X_j^{\beta_j},$$

where v_r is the retropulsion velocity, X_j are the five non-dimensional predictors, and β_j are fitted exponents.
Taking natural logs linearizes the model:

$$\ln v_r = \ln A + \sum_{j=1}^{5} \beta_j \ln X_j + \varepsilon.$$

In this additive log-space, each term $\beta_j \ln X_j$ is a natural contribution to $\ln v_r$. That makes a contribution-analysis in log-space both mathematically consistent and physically meaningful: percent contributions express the relative share of each log-term in producing the predicted log-response.
After fitting this model by least-squares regression (`MATLAB regress`), the contribution of each dimensionless parameter X_j was quantified from the magnitude of its additive log-term:

$$T_{i,j} = \left|\beta_j \ln X_{j,i}\right|.$$

For each predictor, the mean magnitude

$$\overline{T}_j = \frac{1}{n} \sum_i T_{i,j}$$

was evaluated and normalized as

$$C_j = 100\,\frac{\overline{T}_j}{\sum_{k=1}^{5} \overline{T}_k},$$

yielding the relative percentage contribution C_j as presented in Sec. 2.4.3 and Figure 3C in the manuscript. This mean-absolute log-term approach is equivalent to a partial-variance decomposition in log-space and provides a physically interpretable measure of how strongly each parameter influences the predicted retropulsion velocity.
Additionally, coefficient 95% confidence intervals were obtained from the linear regression (`MATLAB regress`), that were used to confirm the robustness of C_j. Group-wise contributions (PN1–3 vs PN4–5) were computed by refitting the log-linear model separately on each pulse-number subgroup and applying the same $T_{i,j} \to \overline{T}_j \to C_j$ procedure.

SI6.3 Residual plot

Figure 13 shows the residual plot. Residuals, defined as the difference between actual and predicted values, are plotted against measured values. The residuals are randomly distributed around zero, indicating no systematic bias and validating the assumption of homoscedasticity. This supports the regression assumption that the variance of the residuals is constant across the range of predicted values.

SI7: Calculations

SI7.1 Uncertainty Estimation for Bubble Growth and Collapse Times

Identifying the growth and collapse times involves tracking the temporal evolution of the bubble width and determining the start, peak, and end times of the oscillation. Ignoring the displacement component of the error propagation (3 μm/pixel, which is much smaller than the typical bubble radius $R_{\max} \sim 1$ mm), the dominant sources of uncertainty arise from identifying (i) the first and last frame corresponding to the start and end of the bubble cycle, and (ii) the frame at which the bubble reaches its maximal size. The uncertainty in the start time originates from jitter in the photodiode output of the laser trigger, which is less than 1 μs. The uncertainty in identifying the frame at which maximal growth is reached is estimated as $\pm\frac{1}{2F}$, where F is the camera frame rate. The same estimate applies for the end frame of collapse.

Since either extreme (early or late frame selection) is equally likely, we apply a statistical combining factor of $1/\sqrt{3}$ for both the maximal-radius and end-frame identifications. This results in a combined uncertainty of

$$\Delta t_c = \sqrt{\left(\frac{1}{\sqrt{3}} \cdot \frac{1}{2F}\right)^2 + \left(\frac{1}{\sqrt{3}} \cdot \frac{1}{2F}\right)^2} = \sqrt{\frac{(12.5)^2}{3} + \frac{(12.5)^2}{3}} = 10.2\ \mu\text{s}.$$

$$\Delta t_g = \sqrt{\left(\frac{1}{\sqrt{3}} \cdot 1\right)^2 + \left(\frac{1}{\sqrt{3}} \cdot \frac{1}{2F}\right)^2} = \sqrt{\frac{(1)^2}{3} + \frac{(12.5)^2}{3}} = 7.2\ \mu\text{s}.$$

The shortest growth time being 175 μs (lowest value was for short pulse in the SD range 0.25-1 mm), the relative error would be ∼4%. With the shortest collapse times being of the order of 165 μs (lowest value was for long pulse in the SD range 0.25-1 mm), the relative error would be 6%. For SD = 0 mm, this would introduce 4% (175 μs, lowest value was for long pulse) and 16.5% (62.5 μs, lowest value was for long pulse)

SI7.2 Added Mass

The added mass is estimated as $m_a = C_m\ \rho_w\ V$, where C_m is a shape-dependent coefficient, $\rho_w = 1000\,\text{kg/m}^3$ is the density of the water, and V is the volume of the object submerged. For a sphere (radius, R_{st}), the added mass would be 50% (C_m=0.5) of the mass of water displaced by it [12, 7]. For the spring, assuming it to be a cylindrical rod – radius R_{sp}, and length, L_{st} – C_m was taken to be 0.8 (instead of 1 [12]) to account for the helical nature of the spring. So the total added mass m_a for this system is :

$$m_a = 0.5 \cdot \left(\rho_w \cdot \frac{4}{3}\pi R_{\text{st}}^3\right) + 0.8 \cdot \left(\rho_w \cdot \pi R_{\text{st}}^2 L_{\text{st}}\right)$$

On inserting the following numbers for the spring (outer radius $R_{\text{sp}} = 2\,\text{mm}$, length $L_{\text{st}} = 25\,\text{mm}$) and the spherical stone ($R_{\text{st}} = 3\,\text{mm}$), the value for m_a may be obtained. Since the spring is attached to the stone, its mass will contribute to the inertia. A correction factor needs to be incorporated for the spring mass ($m_{sp,c}$) as all of (m_{sp}) does not contribute during the impulsive loading from the bubble. Taking the spring to be a cantilever rod (Figure 1), we used inertia from the first mode of vibration as a correction factor. This makes $m_{sp,c} = 0.243 \cdot m_{sp}$. Using these, we can find the ratio of inertias in water vs. air as:

$$\frac{m_{\text{st}} + m_{sp,c} + m_a}{m_{\text{st}} + m_{sp,c}} = 1.58$$

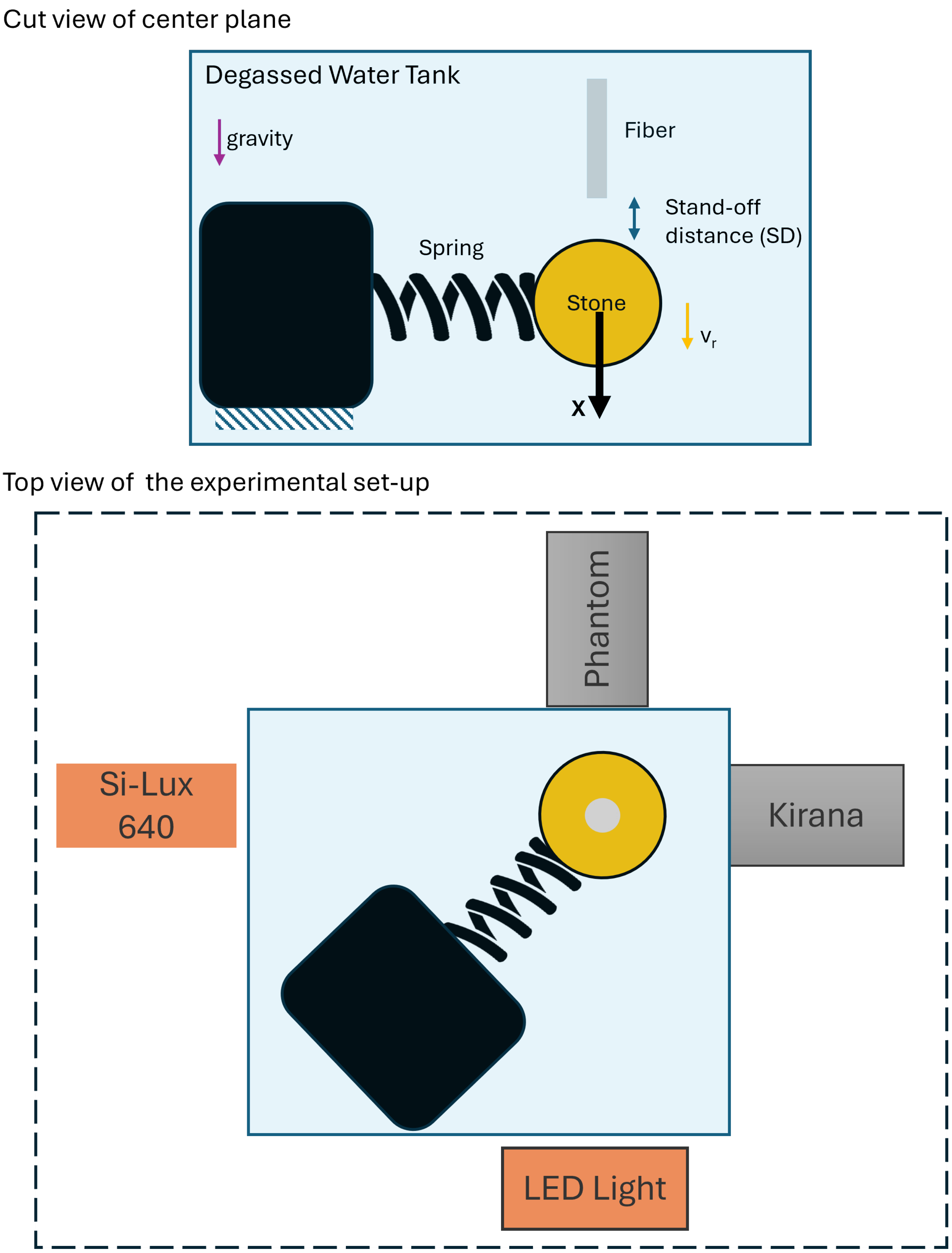

Figure 1: A schematic diagram of the experimental setup, and the camera and light source arrangements. Figure not to scale. The R_{st} = 3 mm. Spring time constant is 96.8 *ms*.

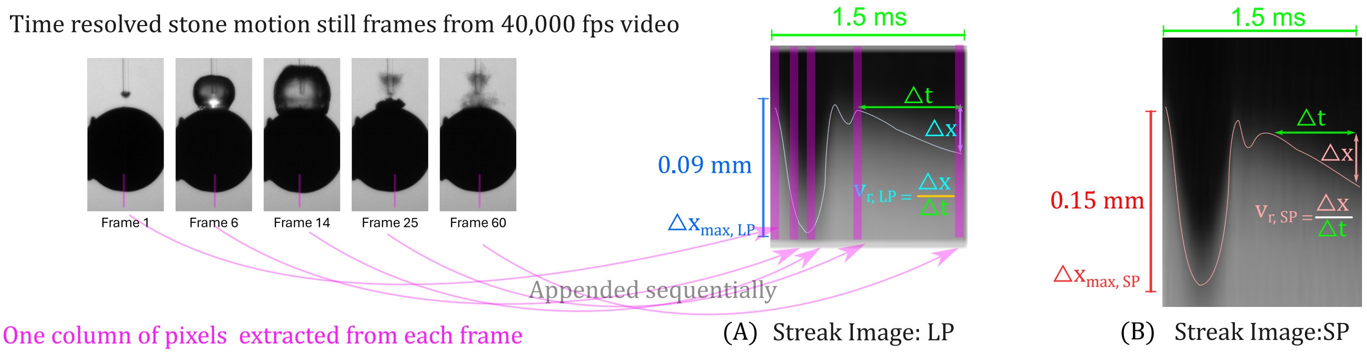

Figure 2: Spatiotemporal streak visualization of stone motion for LP (A) and SP (B) cases, whose displacement curves were extracted and shown in Fig.1. Theses composed images were constructed by extracting a single vertical column of pixels along the central axis of the stone at each frame and appending them sequentially in time. This approach converts the temporal evolution of motion into a two-dimensional space–time map, that is convenient to visualize stone retropulsive motion. Each image was stitched together from 60 frames, representing a total time window of 1.5 ms. The vertical dimension corresponds to the measured displacement range (Δx_{max}) from Fig. 1A. The downward slope in each image indicates the direction and magnitude of stone motion away from the laser fiber and their slopes (annotated in these images) correspond to the stone retropulsion velocities (v_r).

Table 1: Crater volumes, V_c (mm^3) for SP and LP treatment modes for SD = 0.5 mm

Pulse type	Wet-Air	Water
Short Pulse (SP)	0.015±0.002	0.012±0.0019
Long Pulse (LP)	0.036±0.004	0.018±0.001

Movie Files

- `Ejecta from stone in air. Long pulse.`
- `Short pulse at SD 0.5 mm, PN1.`
- `Long pulse at SD 0.5 mm, PN1.`
- `Ejecta escaping the bubble, Short Pulse, SD 0.5 mm, PN3.`
- `Ejecta escaping the bubble, Short Pulse, SD 0.5 mm, PN4.`

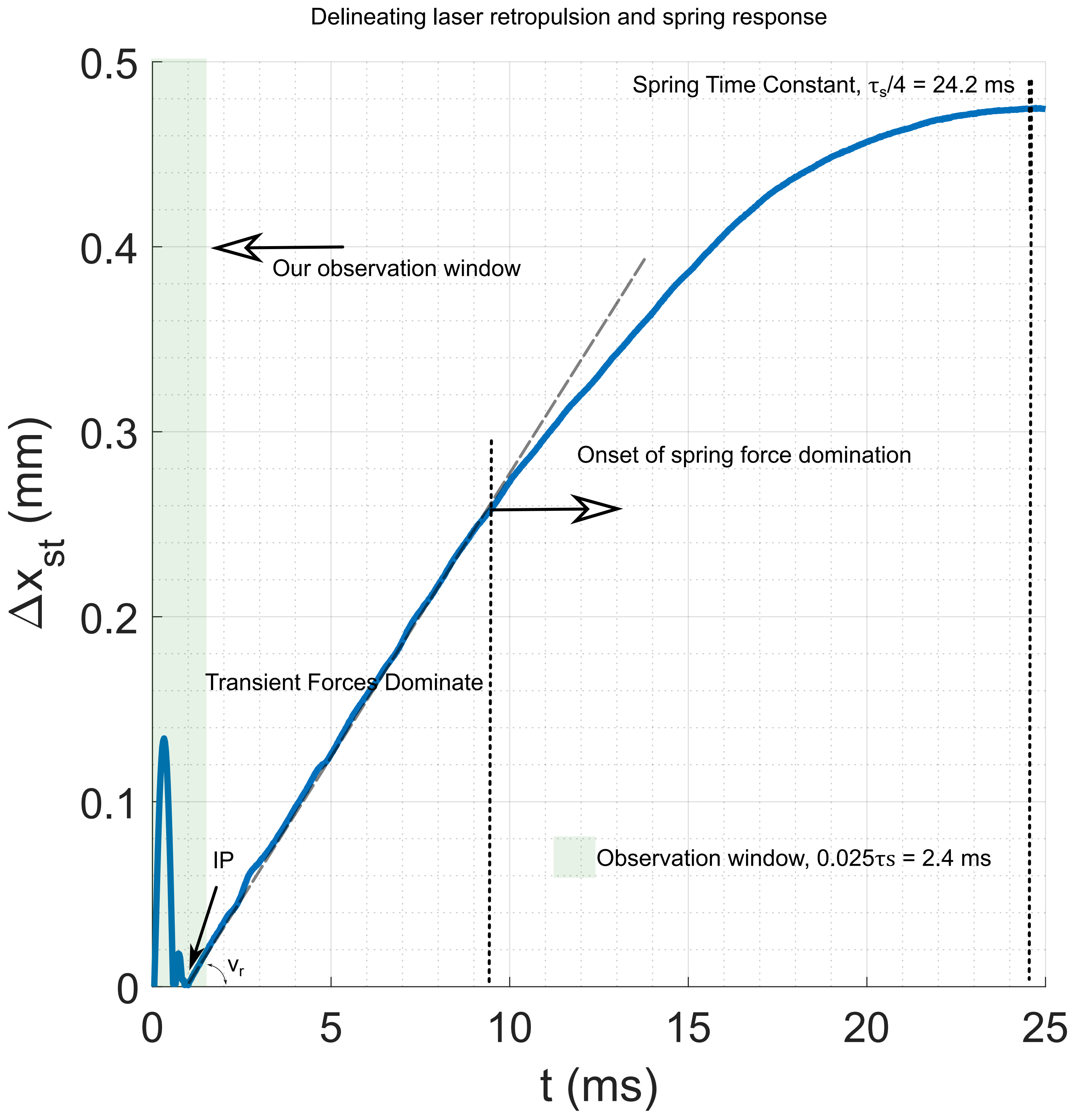

Figure 3: Displacement curve for the stone for durations longer than our observation window. The spring force comes into action from about 10 ms. The inflection point (IP) and the region where we measure stone retropulsion (v_r), may be seen to be within the region where the spring force is minimal.

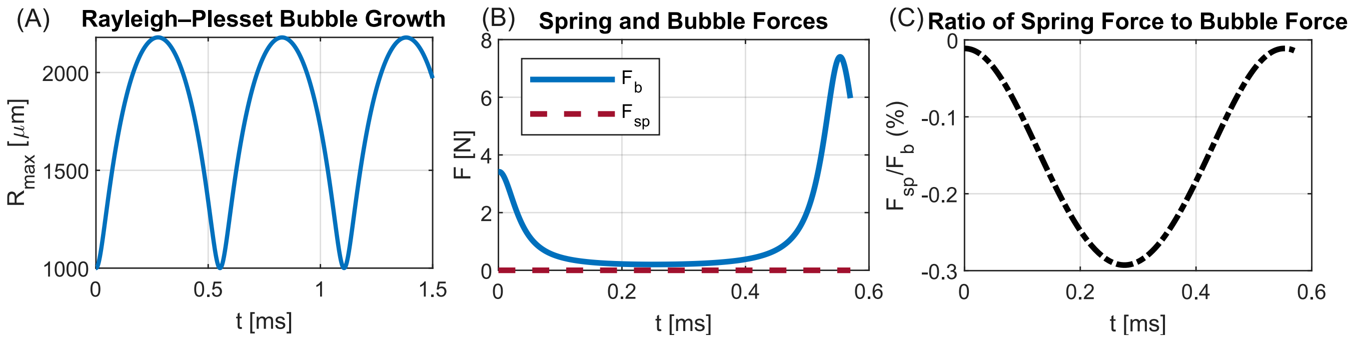

Figure 4: (A) Bubble radius as a function of time, using Raleigh-Plesset equation. (B) Estimated Spring and Bubble Forces (C) Percentage of the Spring Force to the Bubble Force.

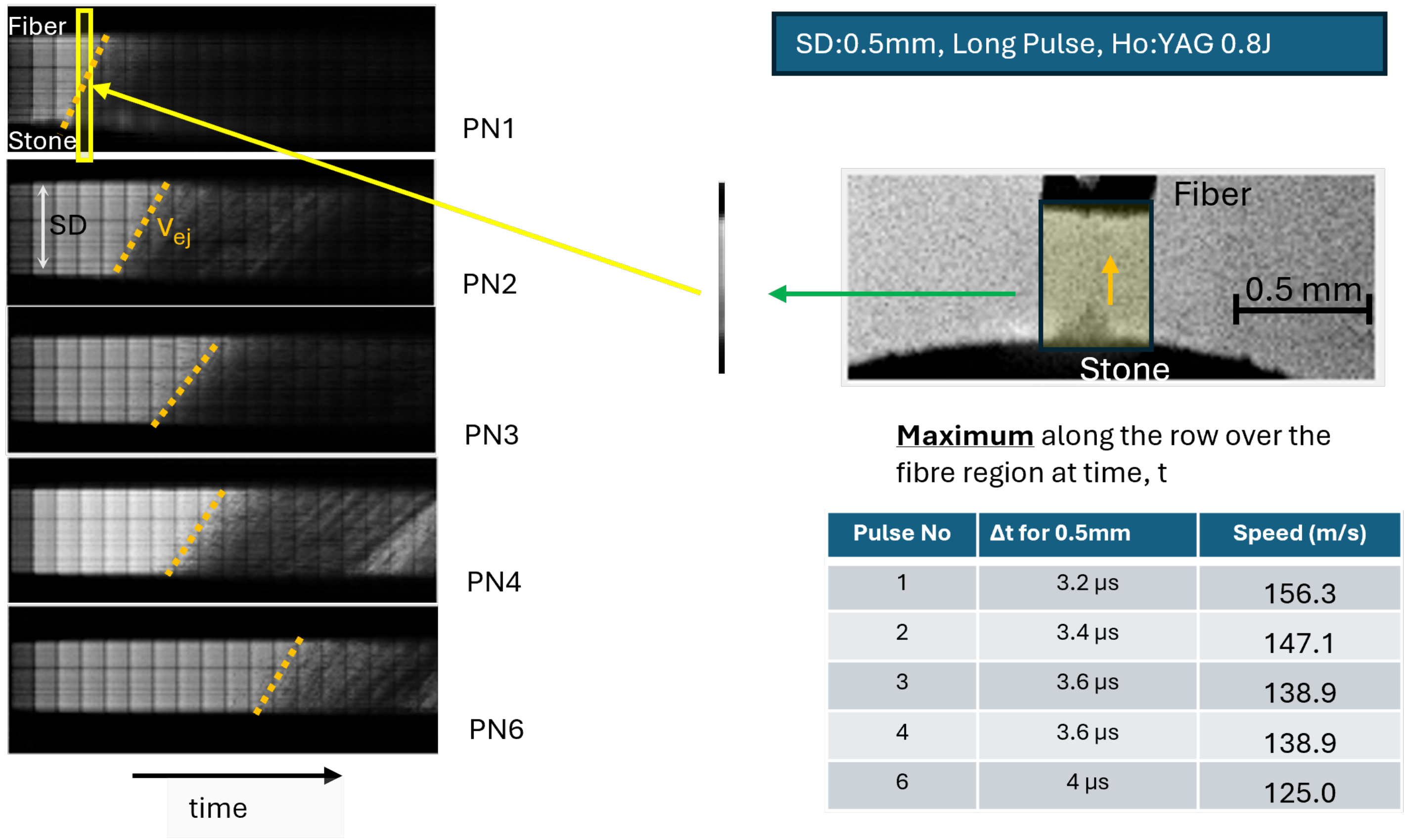

Figure 5: Ejecta velocity measurement methodology using high-speed imaging. (Left) Raw high-speed images at 5 Mfps showing stone surface ablation and ejecta formation. The fiber (top) and stone surface (bottom) are clearly visible. (Center) Synthetic streak images generated by extracting maximum intensity pixels along the fiber axis for different pulse numbers (PN1-PN6). The diagonal streaks represent ejecta trajectories. (Right) Quantitative ejecta velocity measurements showing decreasing speed with pulse number due to crater deepening and reduced laser fluence. Scale bar: 0.5 mm. Error bars represent standard deviation of n=4 measurements.

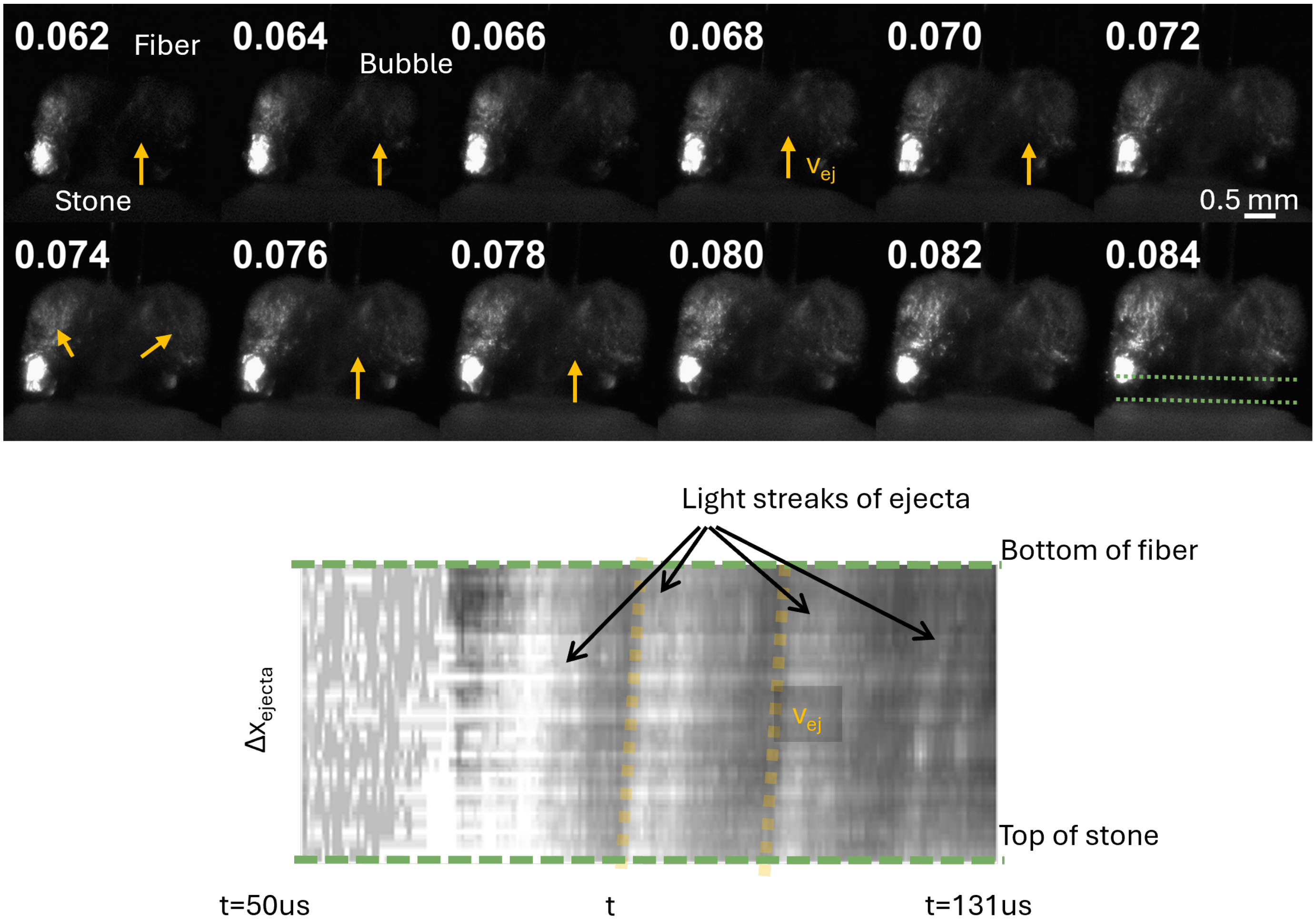

Figure 6: Top: High-speed images of bubble-ejecta dynamics; yellow arrows indicate ejecta presence. Bottom: A synthetic image obtained by extracting pixels along the line drawn from the top of the stone to the bottom of the fiber, along the fiber's central axis. These time resolved pixel "lines" were placed next to each other. The ejecta leaving the stone surface are seen as a series of squirts from the surface of the stone. The slope of this trajectory would provide the velocity of the ejecta. The speeds are fairly constant for each squirt. The time intants are in *ms*.

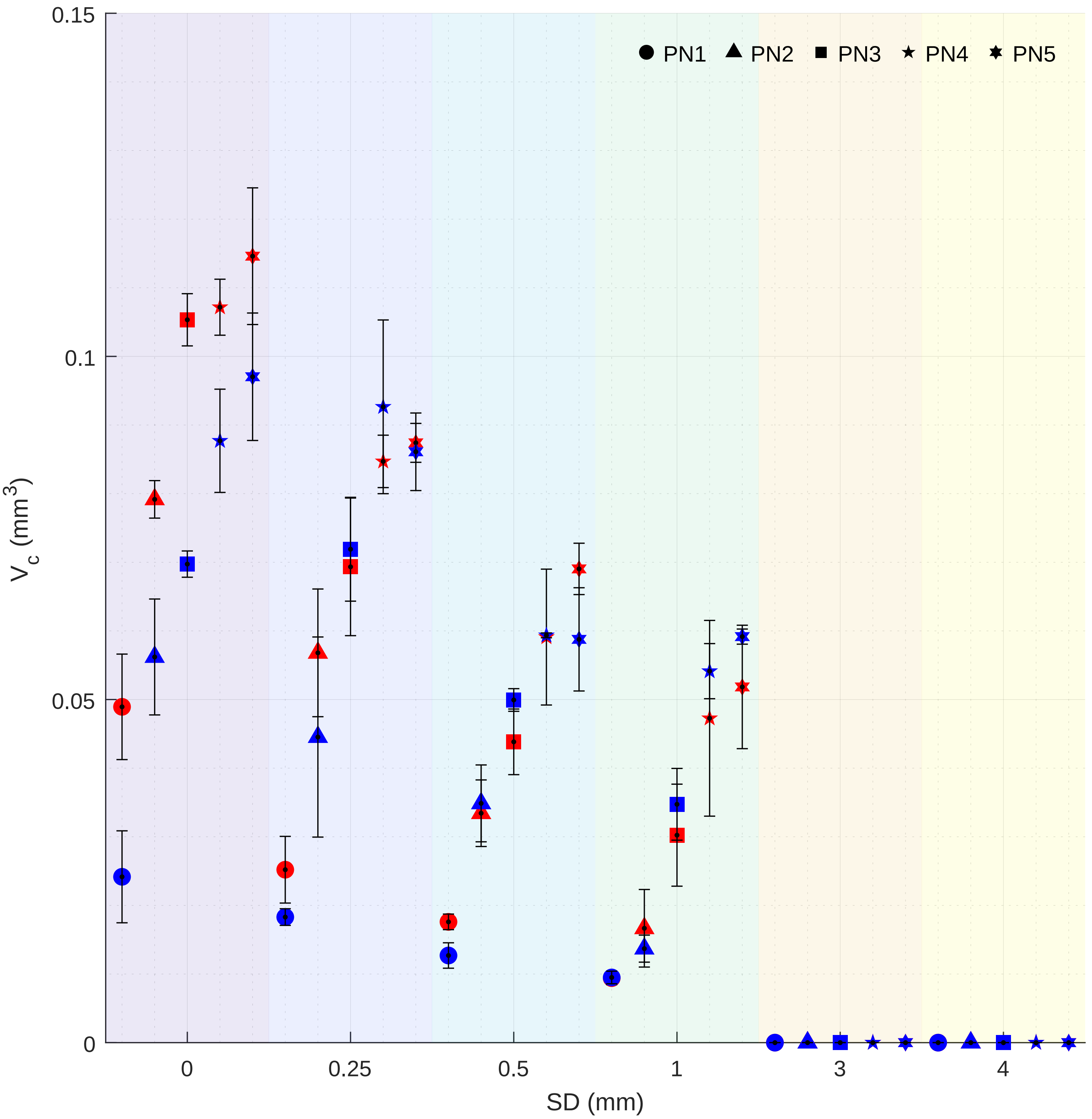

Figure 7: Crater volume variation for each stand-off distance.

Table 2: Velocity Data for long pulse (LP) and short pulses (SP) with standard deviations σ

Pulse	Stand-off Distance (mm)					
Number	0	0.25	0.5	1	3	4
			$v_{r,LP} \pm \sigma$ (mm/s)			
PN 1	73.72 ± 11.45	24.78 ± 1.67	17.69 ± 0.88	11.24 ± 0.90	4.97 ± 4.42	2.25 ± 0.37
PN 2	66.64 ± 3.06	30.51 ± 3.40	21.32 ± 2.14	10.18 ± 2.13	4.97 ± 4.42	2.25 ± 0.37
PN 3	76.59 ± 6.97	39.33 ± 4.65	24.54 ± 1.16	8.79 ± 2.89	4.97 ± 4.42	2.25 ± 0.37
PN 4	85.00 ± 5.34	41.87 ± 4.12	27.80 ± 1.34	9.51 ± 2.65	4.97 ± 4.42	2.25 ± 0.37
PN 5	94.93 ± 5.82	45.82 ± 3.24	29.54 ± 3.94	8.95 ± 2.45	4.97 ± 4.42	2.25 ± 0.37
			$v_{r,SP} \pm \sigma$ (mm/s)			
PN 1	71.75 ± 8.01	44.45 ± 7.48	40.46 ± 4.94	27.30 ± 2.77	2.39 ± 1.74	3.47 ± 2.40
PN 2	56.07 ± 2.48	40.58 ± 4.15	36.02 ± 5.62	27.95 ± 3.16	2.39 ± 1.74	3.47 ± 2.40
PN 3	59.64 ± 5.03	46.63 ± 2.03	37.53 ± 7.50	26.78 ± 0.98	2.39 ± 1.74	3.47 ± 2.40
PN 4	72.00 ± 5.03	52.08 ± 6.50	41.23 ± 5.94	26.55 ± 2.77	2.39 ± 1.74	3.47 ± 2.40
PN 5	82.06 ± 5.63	50.97 ± 5.80	41.41 ± 6.56	23.18 ± 3.32	2.39 ± 1.74	3.47 ± 2.40

Table 3: p-values (rounded to 2 decimals) for pairwise comparisons of v_r values from consecutive pulse numbers (PNs). A p-value < 0.05 indicates a statistically significant difference in velocity. p-values are rounded to two decimal places.

Comparison	Stand-off Distance (mm)					
	0	0.25	0.5	1	3	4
			Long Pulse			
PN1 vs PN2	0.02	0.00	0.00	0.09	0.39	0.67
PN2 vs PN3	0.00	0.00	0.02	0.24	0.15	0.52
PN3 vs PN4	0.01	0.47	0.00	0.90	0.52	0.32
PN4 vs PN5	0.00	0.00	0.97	0.67	0.81	0.05
			Short Pulse			
PN1 vs PN2	0.00	0.01	0.08	0.85	0.55	0.21
PN2 vs PN3	0.27	0.00	0.26	0.59	0.47	0.42
PN3 vs PN4	0.00	0.08	0.31	0.97	0.14	0.64
PN4 vs PN5	0.01	0.97	0.17	0.09	0.77	0.05

Table 4: p-values (rounded to 2 decimals) for v_r comparison for each pulse number against PN1. A p-value < 0.05 indicates a statistically significant difference in velocity. p-values are rounded to two decimal places.

Comparison	Stand-off Distance (mm)					
	0	0.25	0.5	1	3	4
			Long Pulse			
PN2 vs PN1	0.02	0.00	0.00	0.09	0.39	0.67
PN3 vs PN1	0.36	0.00	0.00	0.01	0.65	0.32
PN4 vs PN1	0.01	0.00	0.00	0.06	0.85	0.95
PN5 vs PN1	0.00	0.00	0.00	0.11	0.65	0.05
			Short Pulse			
PN2 vs PN1	0.00	0.01	0.08	0.85	0.55	0.21
PN3 vs PN1	0.00	0.82	0.63	0.68	0.97	0.10
PN4 vs PN1	0.80	0.17	0.41	0.77	0.21	0.14
PN5 vs PN1	0.03	0.15	0.42	0.05	0.28	0.01

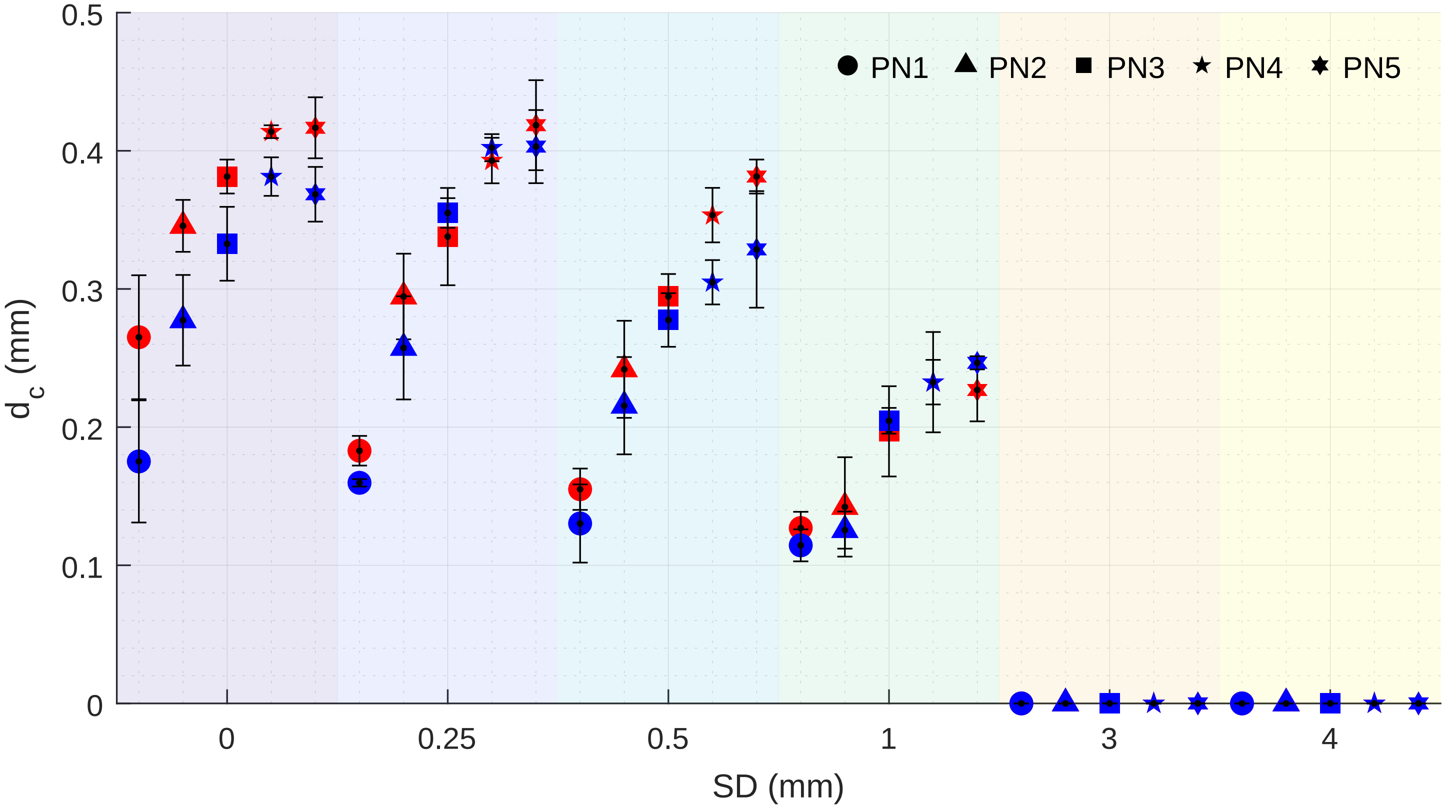

(a) Crater depth, d_c

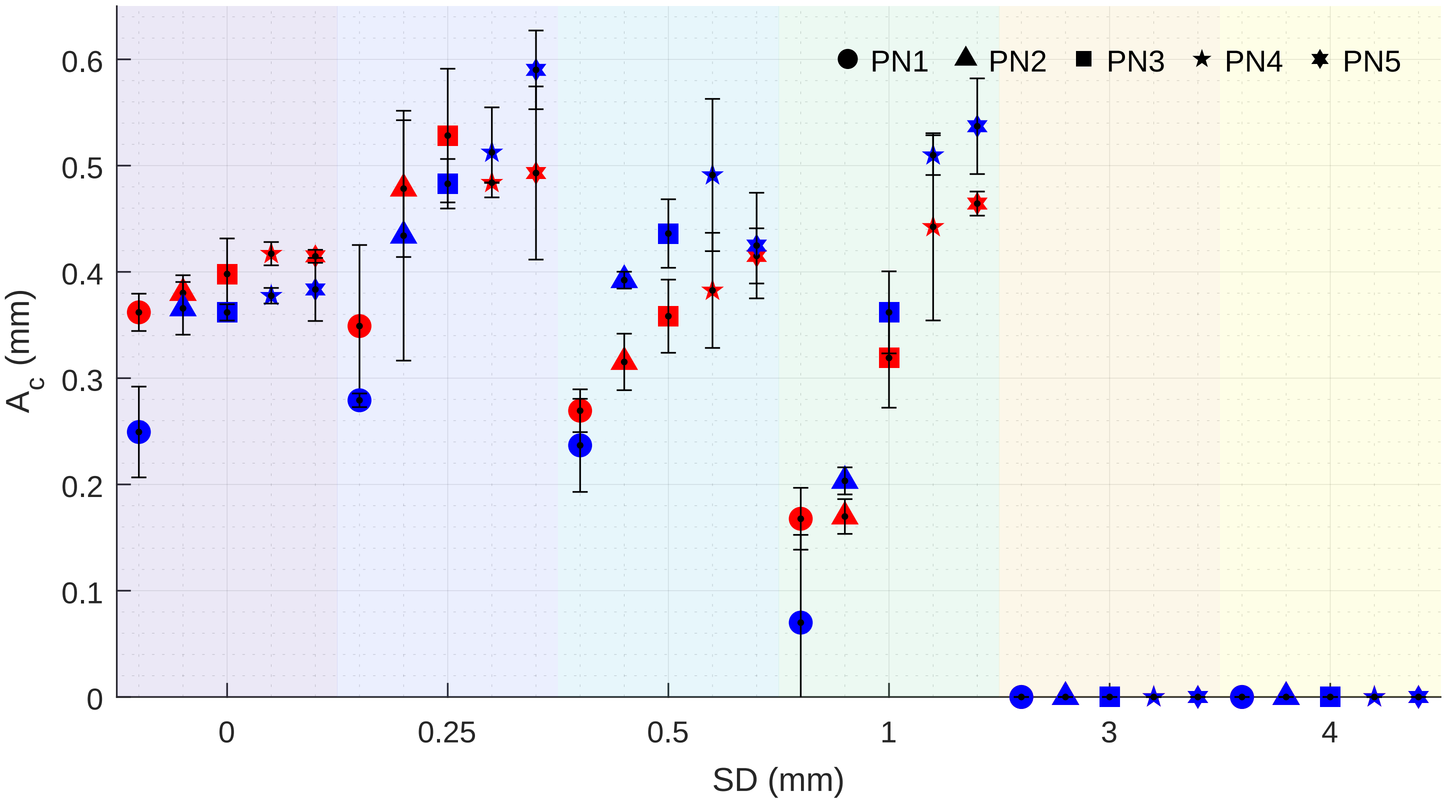

(b) Crater surface area, A_c

Figure 8: Crater depth and surface area variation from pulse to pulse and for different stand-off distances

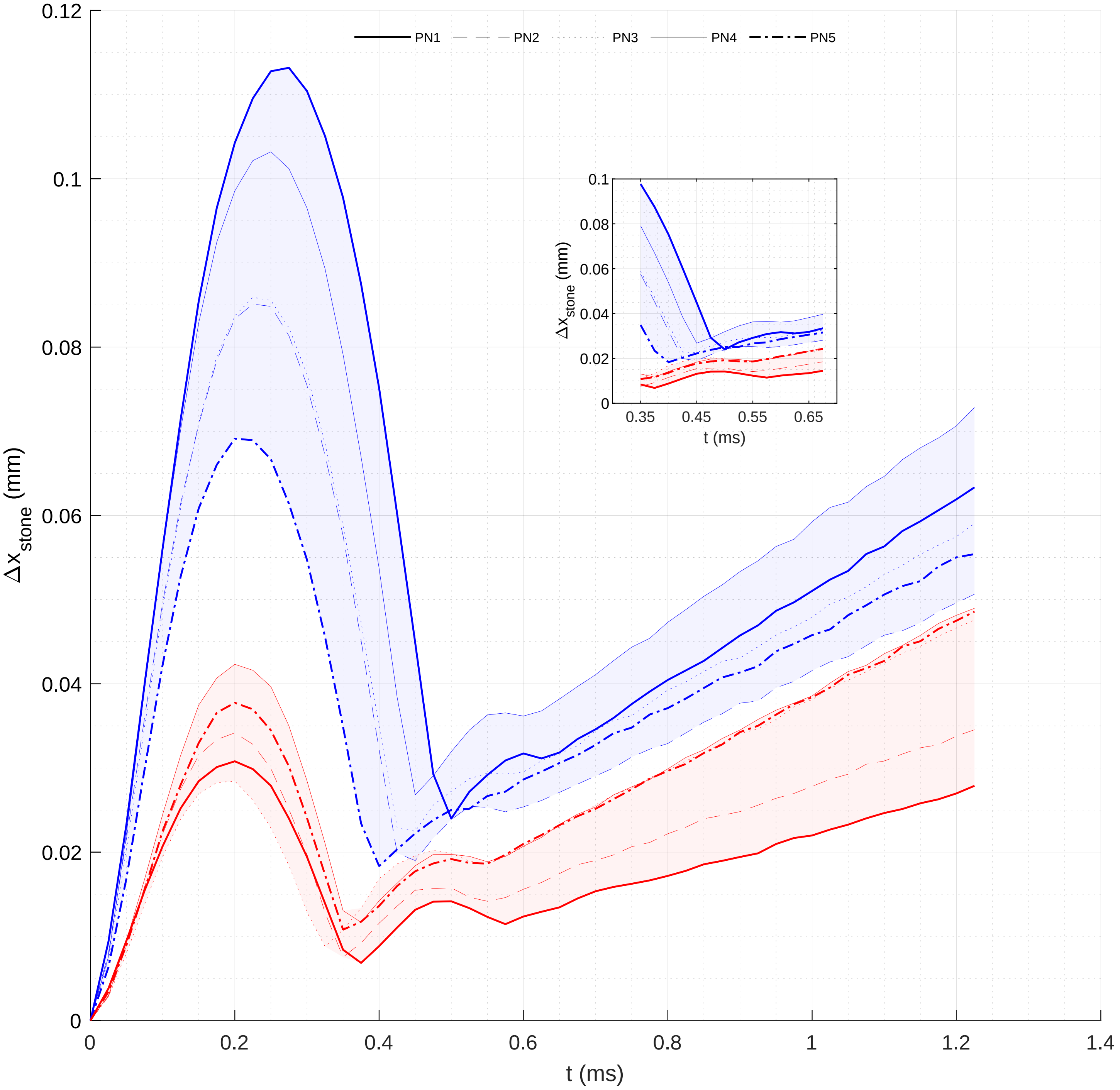

Figure 9: Displacement for SD 0.25 mm, for 5 PNs

Table 5: Characteristic timescales of processes involved in retropulsion

**Process**	**Timescale (ms)**	**Effect**
Laser Pulse (Short, FWHM = 80 μs)	0.15	Energy input
Laser Pulse (Long, FWHM = 205 μs)	0.25	Energy input
Ejecta Burst	0.25	Modulation of bubble
Bubble Collapse	0.80	Primary momentum transfer
**Spring Response**	**96.8**	**Post-impulse oscillation only**

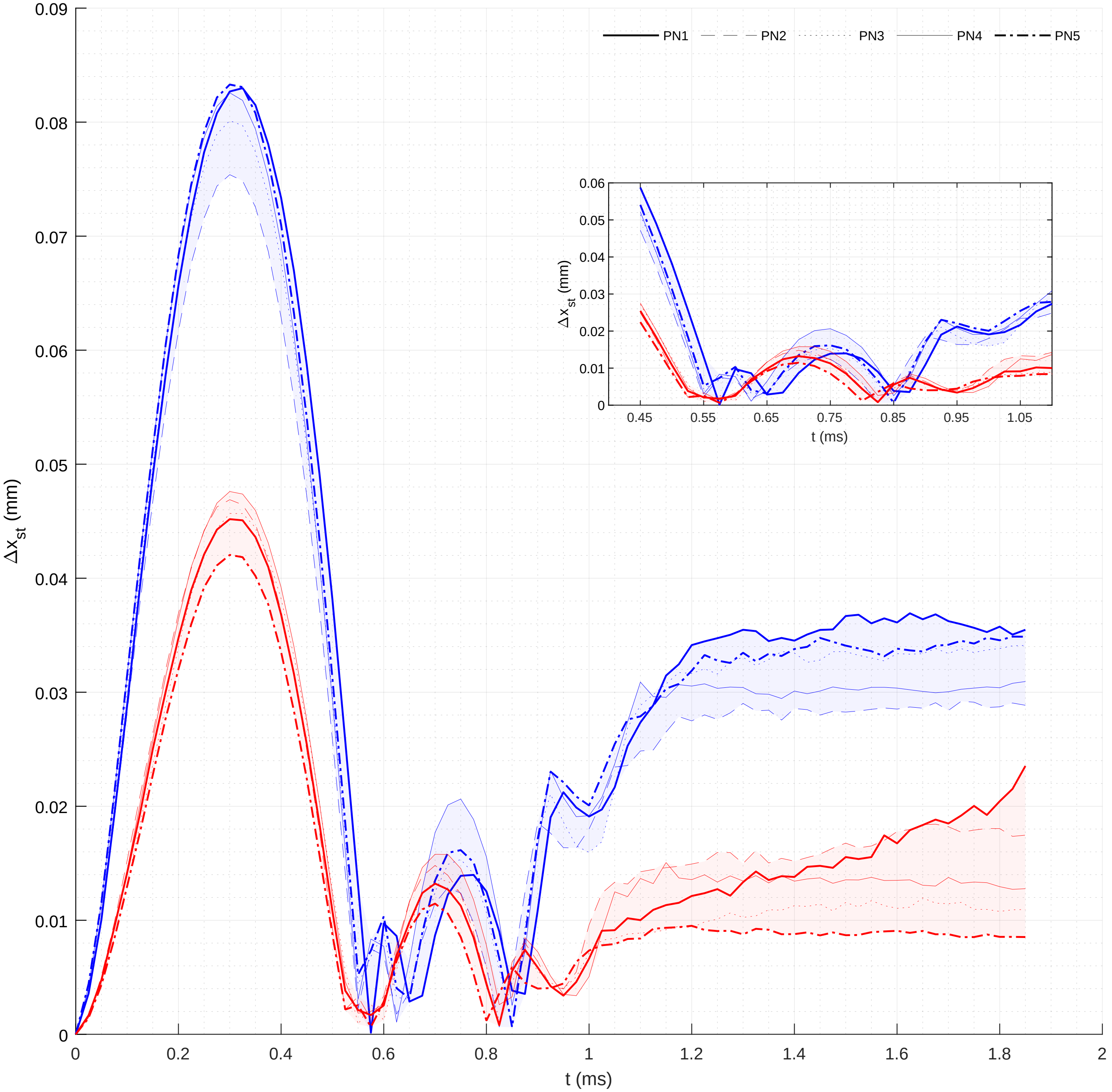

Figure 10: Stone displacement data for SD 3mm, for 5 repeats.

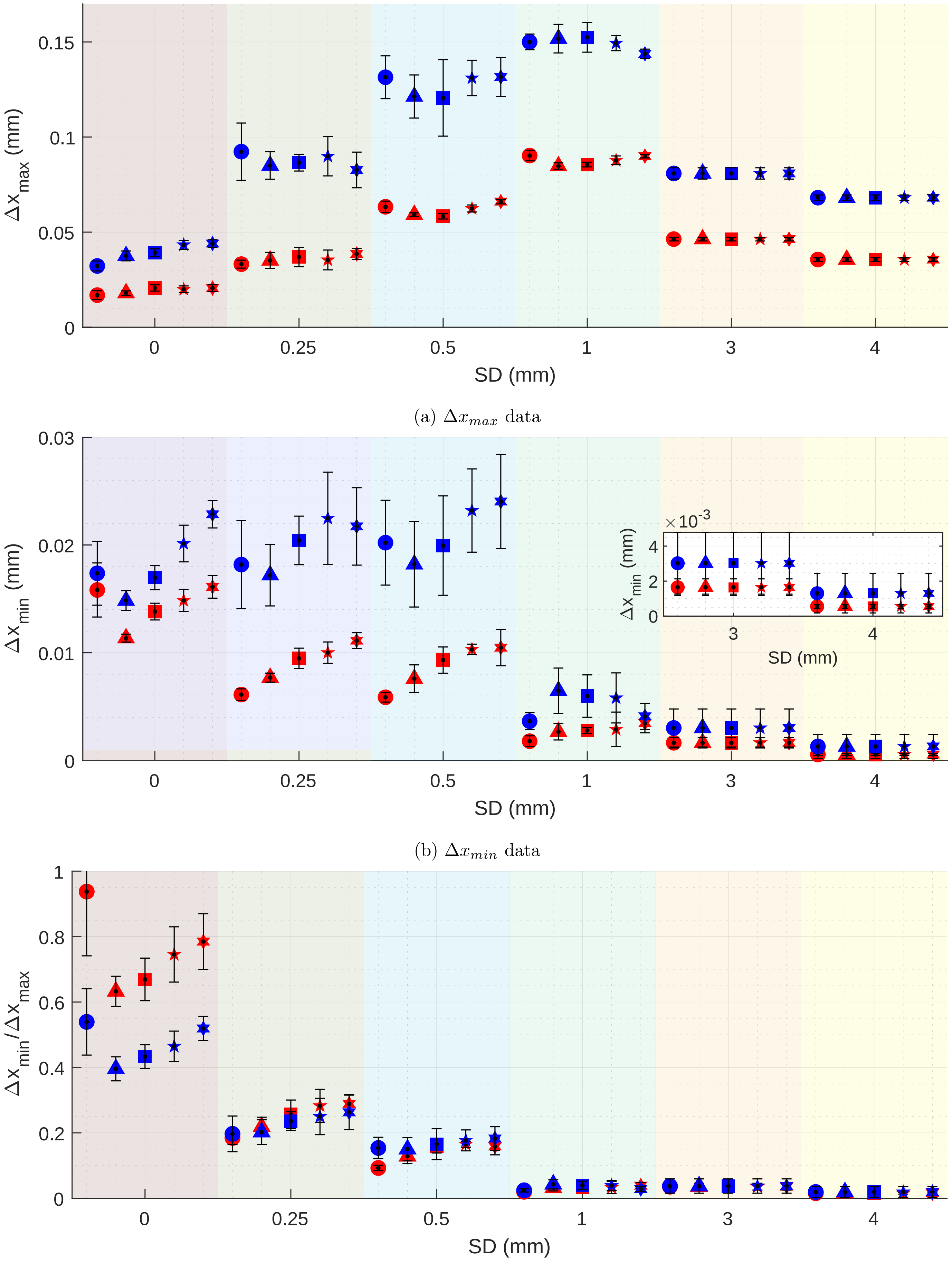

(a) Δx_{max} data

(b) Δx_{min} data

(c) $\Delta x_{min}/\Delta x_{max}$ data

Figure 11: Combined plots of parameters from the stone displacement curves as a function of SD.

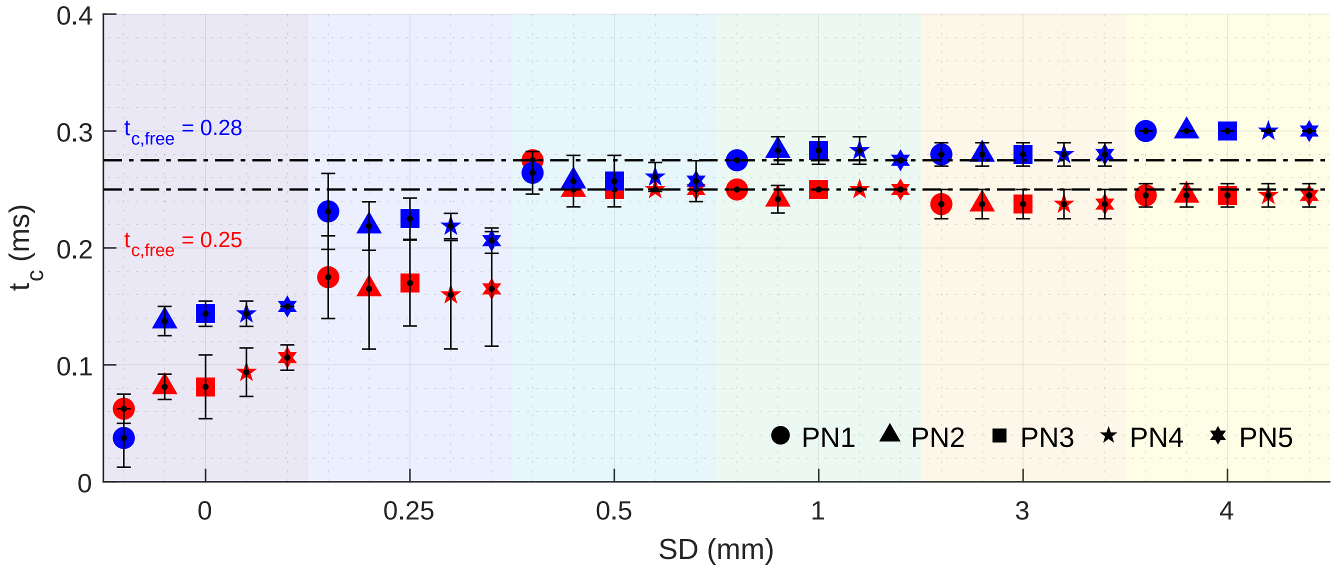

(a) Bubble collapse time, t_c

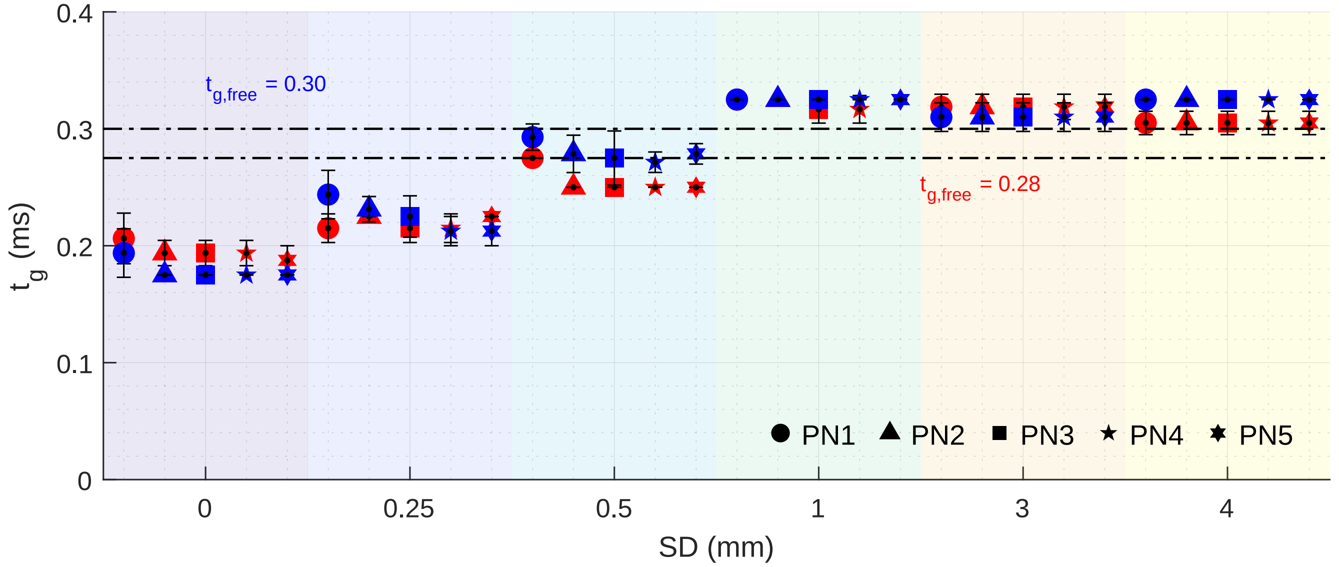

(b) Bubble growth time, t_g

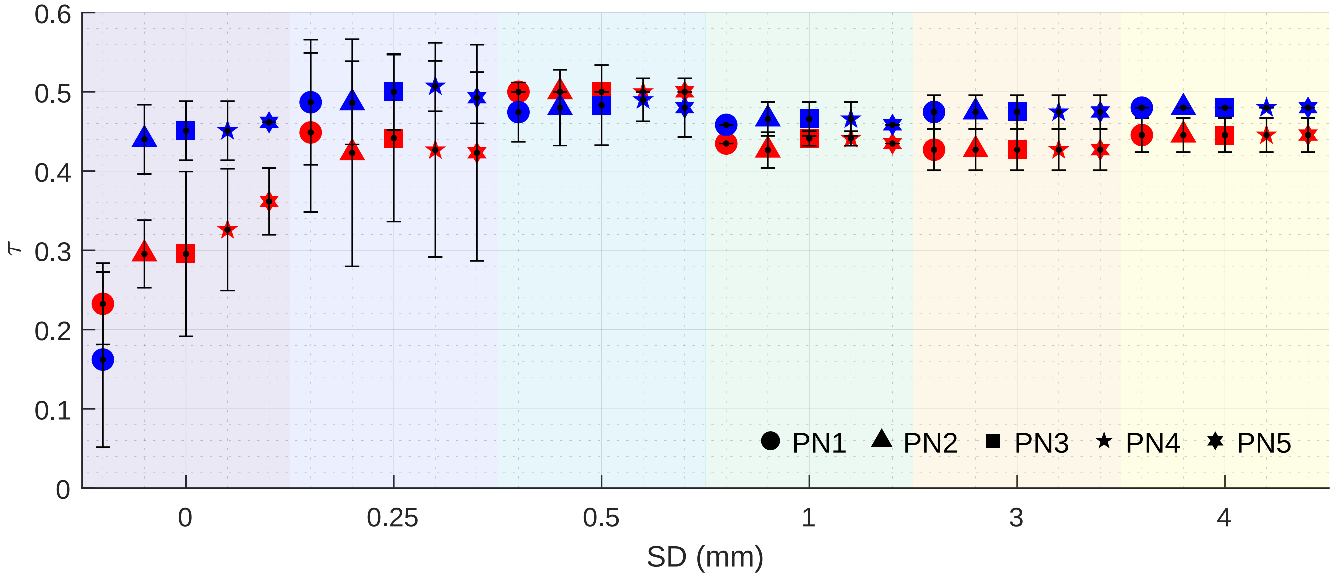

(c) τ variation with SD.

Figure 12: Variation of collapse times (t_c), growth times (t_g), and $\tau = t_c/(t_c + t_g)$ with SD. Note the peak at SD 0.25mm for short pulse, and at SD 0.5 mm for long pulse.

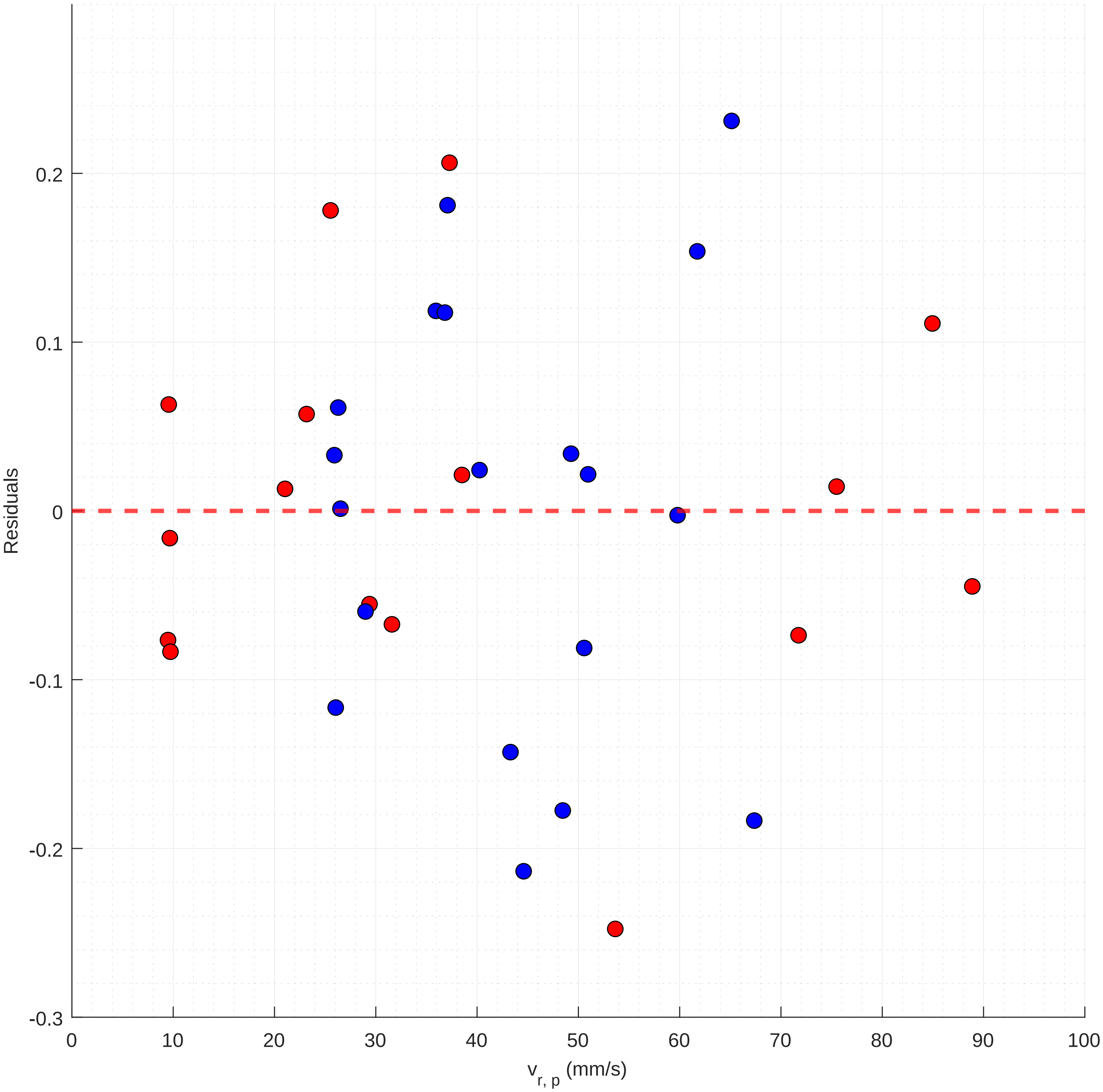

Figure 13: Residual plot for model validation as shown in Figure 3

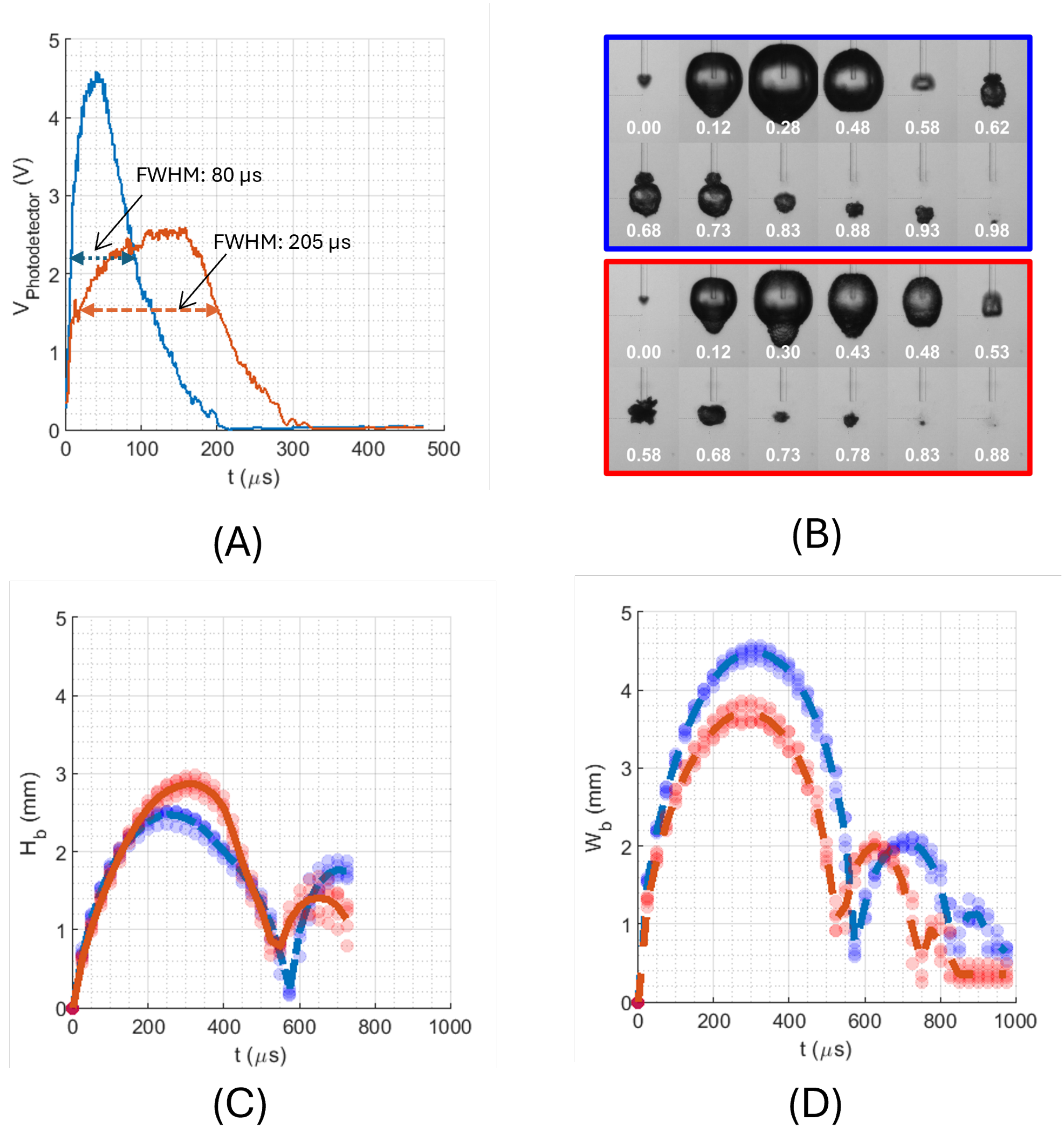

Figure 14: (A) The pulse profile for short pulse (blue, FWHM = 80 μs) and long pulse (red, FWHM = 205 μs). (B) The resultant free-field evolution of the bubble for short pulse (top, blue outline) and long pulse (bottom, red outline) is shown at instants in ms. (C) Evolution of bubble length, L_b, and (D) bubble width, W_b, in the free-field.

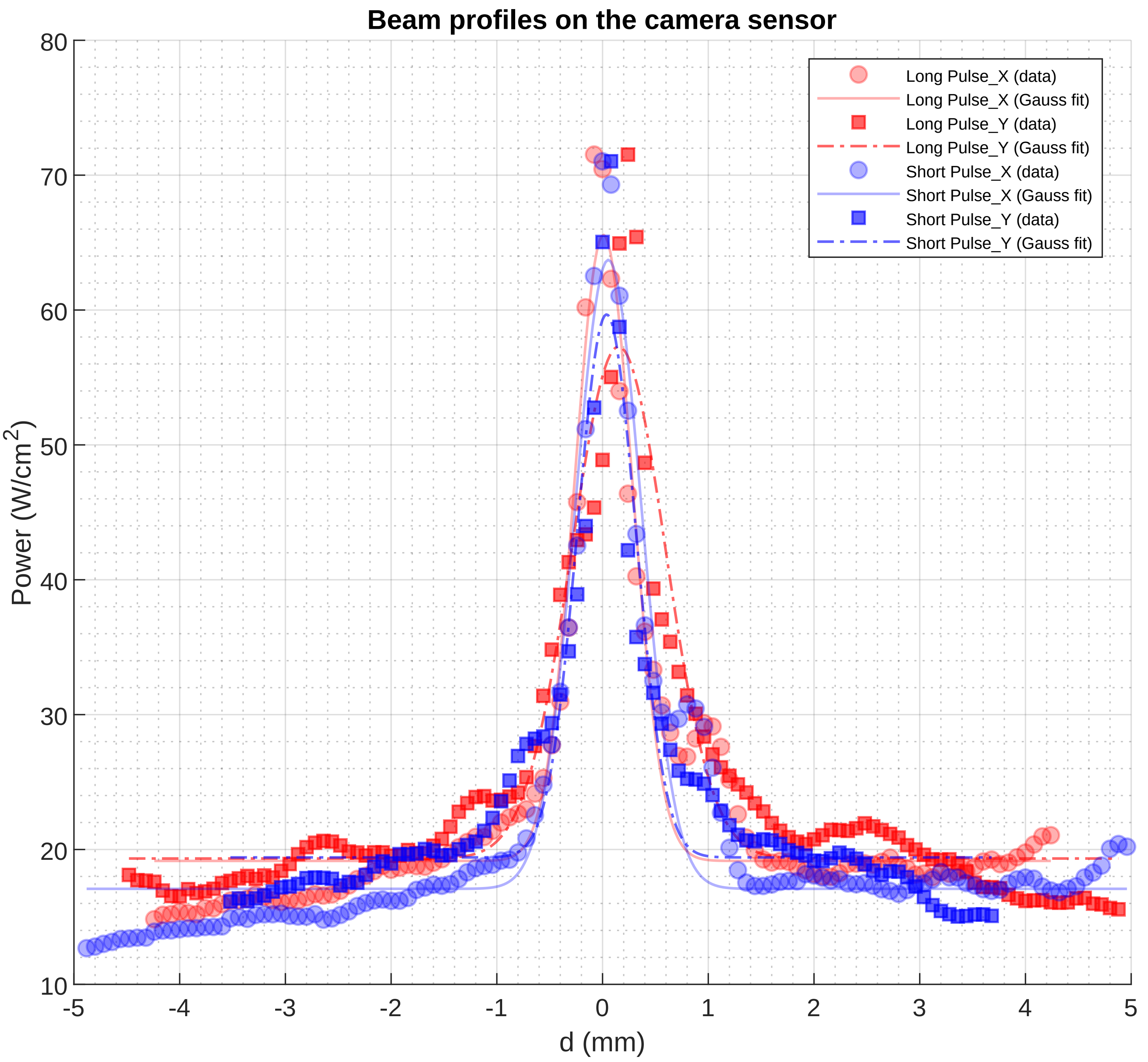

Figure 15: Beam profiles as observed on the camera sensor. The R^2 was 0.91 and 0.90 for LP along X and Y-axis, respectively. And for SP, it was 0.92 and 0.88.

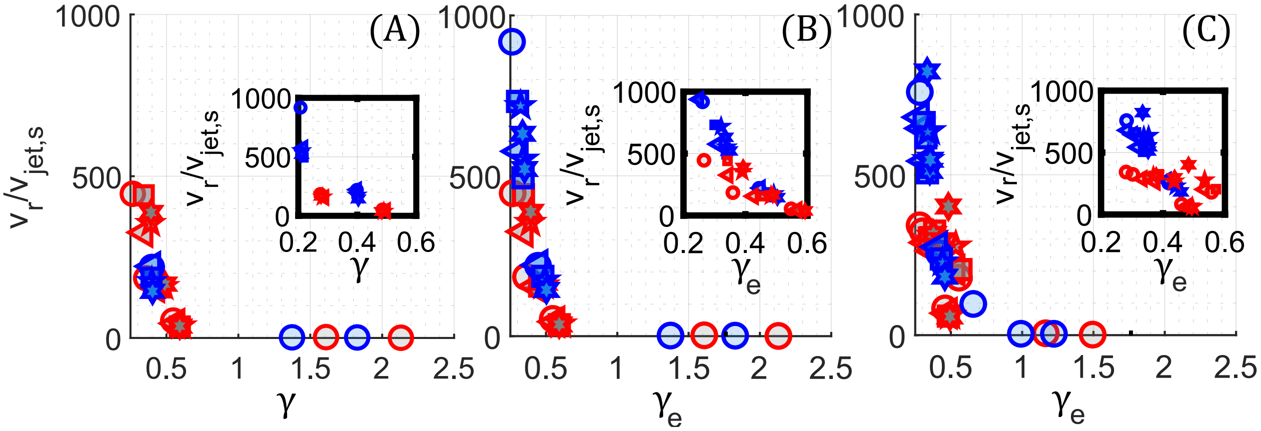

Figure 16: Comparison of different retropulsion data normalization approaches. Each panel plots the measured normalized retropulsion velocity v_r/v_{jet}(Markers represent pulse numbers, and the colors correspond to the type of the pulse) versus the effective stand-off distances. Insert shows a zoom onto the range 0.2 < γ_e < 0.6. (A) Original Outi et al. (2016) [11] formulation using the nominal stand-off SD and the classical $v_{jet} \propto \gamma^{-2}$ scaling. The original model (planar, stationary wall assumption) fails to collapse the dataset: large systematic scatter and offsets are evident. (B) Outi [11] scaling modified by the pulse-dependent effective stand-off SD_e=SD+d_c, where d_c is the crater depth measured by OCT after each pulse. Replacing SD with SD_e substantially reduces scatter and brings different pulse conditions into better agreement, demonstrating the first-order role of crater confinement. (C) Curvature correction (Tomita et al. [14]) applied to the SD_e-normalized model: the collapse time is modified by the curvature-dependent prolongation factor $\mu(R_{st})$ and the jet speed is corrected accordingly ($v_{(jet,corr)} \propto R_{max}/(\mu(R_{st})t_R)$). Including curvature yields the tightest collapse across the dataset, especially at small γ_e, showing that finite stone curvature and crater geometry are essential to quantitatively predict jet speed in our experiments. Insets: zoom of low− γ_e region where the corrections have the largest effect.

Table 6: Summary of hydrophone measurements showing the peak pressure: $p_{\max}$, time at which peak pressure occurs: $t_{\max}$, peak pressure in the 100 μs window before inflection point: $p_{IP-100\mu s}$, time at which $p_{IP-100\mu s}$ occurs: $t_{IP-100\mu s}$, and the time at which inflection point (IP) occurs: t_{IP}. Values are given as mean±SD rounded to 2 decimals; parenthesized p-values (rounded to 2 decimals) are provided where necessary. Paired two tail t-test was used to compute p-value.

	$p_{\max}$ (MPa)		$t_{\max}$ (ms)	$p_{IP-100\mu s}$ (MPa)		$t_{IP-100\mu s}$ (ms)	t_{IP} (ms)
**SD 0.25 mm — Long Pulse (n = 5)**							
PN1	0.54 ± 0.34		0.36 ± 0.04	0.08 ± 0.04		0.52 ± 0.05	0.56 ± 0.02
PN2	0.80 ± 0.30	(p = 0.06)	0.31 ± 0.01	0.11 ± 0.10	(p = 0.44)	0.49 ± 0.04	0.54 ± 0.03
PN3	0.91 ± 0.34	(p = 0.30)	0.32 ± 0.02	0.11 ± 0.05	(p = 0.91)	0.44 ± 0.03	0.53 ± 0.03
PN4	1.08 ± 0.20	(p = 0.11)	0.32 ± 0.01	0.14 ± 0.12	(p = 0.38)	0.50 ± 0.03	0.55 ± 0.02
PN5	1.07 ± 0.17	(p = 0.86)	0.32 ± 0.02	0.28 ± 0.35	(p = 0.45)	0.46 ± 0.09	0.51 ± 0.07
**SD 0.25 mm — Short Pulse (n = 10)**							
PN1	1.63 ± 0.81		0.42 ± 0.03	0.30 ± 0.15		0.58 ± 0.17	0.64 ± 0.15
PN2	1.23 ± 0.63	(p = 0.02)	0.39 ± 0.03	0.24 ± 0.10	(p = 0.14)	0.49 ± 0.06	0.56 ± 0.04
PN3	1.76 ± 0.80	(p = 0.00)	0.39 ± 0.04	0.26 ± 0.11	(p = 0.56)	0.49 ± 0.06	0.57 ± 0.05
PN4	1.71 ± 0.51	(p = 0.86)	0.39 ± 0.06	0.28 ± 0.12	(p = 0.49)	0.50 ± 0.06	0.56 ± 0.06
PN5	2.01 ± 0.96	(p = 0.10)	0.40 ± 0.05	0.60 ± 0.91	(p = 0.32)	0.50 ± 0.05	0.56 ± 0.05
**SD 3 mm (n = 20)**							
LP	0.53 ± 0.23		0.52 ± 0.07	0.05 ± 0.02		1.10 ± 0.26	1.13 ± 0.24
SP	2.56 ± 0.74		0.40 ± 0.03	0.06 ± 0.02		1.16 ± 0.17	1.20 ± 0.17
**SD 4 mm (n = 21–22)**							
LP	0.60 ± 0.16		0.49 ± 0.08	0.05 ± 0.02		1.03 ± 0.24	1.07 ± 0.23
SP	3.55 ± 0.57		0.39 ± 0.02	0.24 ± 0.76		1.08 ± 0.22	1.13 ± 0.20

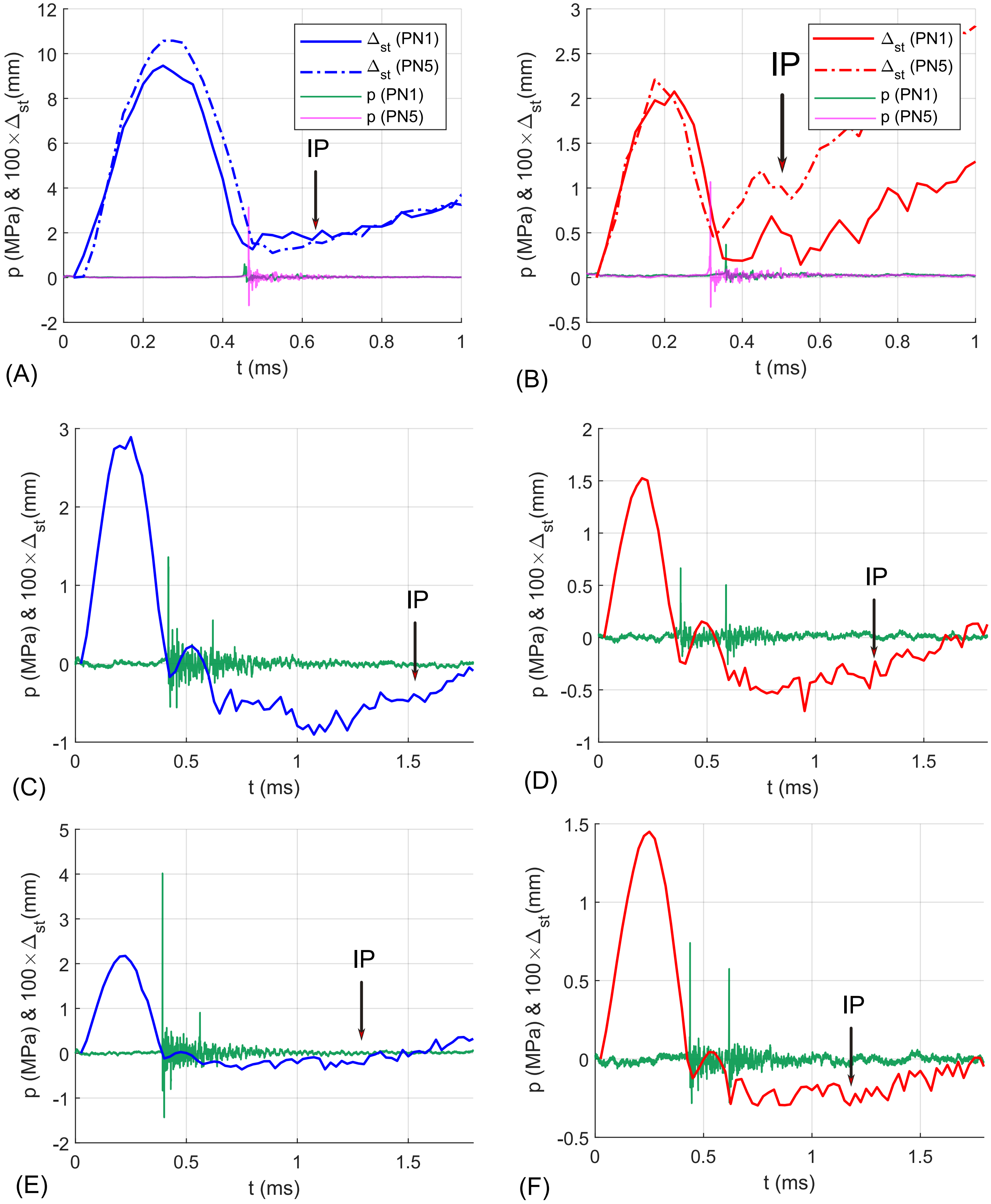

Figure 17: Typical stone displacement and corresponding corrected hydrophone data. The short pulse displacement data is shown in blue and the long pulse displacement data in red. PN1 is provided with a bold line and PN5 has a dashed line where applicable. Also, for pressure, dark green is PN1 and magenta in PN5. Top row: SD = 0.25 mm, SP (A) and LP (B), Middle row: SD = 3 mm, SP (D) and LP (E), Last row: SD= 4 mm, SP (F) and LP (G). The location of the inflection point (IP) has been shown in each plot.

References

[1] Brennen, C.E.: Cavitation and bubble dynamics. Cambridge university press (2014)

[2] Cummings, J.P., Walsh, J.T.: Tissue tearing caused by pulsed laser-induced ablation pressure. Applied Optics 32(4), 494–503

[3] Esch, E., Simmons, W.N., Sankin, G., Cocks, H.F., Preminger, G.M., Zhong, P.: A simple method for fabricating artificial kidney stones of different physical properties. Urological Research 38(4), 315–319 (2010)

[4] Fried, N.M.: Recent advances in infrared laser lithotripsy. Biomedical Optics Express 9(9), 4552–4568 (2018)

[5] Ho, D.S., Scialabba, D., Terry, R.S., Ma, X., Chen, J., Sankin, G.N., Xiang, G., Qi, R., Preminger, G.M., Lipkin, M.E., et al.: The role of cavitation in energy delivery and stone damage during laser lithotripsy. Journal of Endourology 35(6), 860–870 (2021)

[6] Lee, H., Ryan, R.T., Teichman, J.M., Landman, J., Clayman, R.V., Milner, T.E., Welch, A.: Effect of lithotripsy on Holmium:YAG optical beam profile. Journal of Endourology 17, 63–67 (2003)

[7] Newman, J.N.: Marine Hydrodynamics. The MIT Press (1977)

[8] Petzold, R., Miernik, A., Suarez-Ibarrola, R.: Retropulsion force in laser lithotripsy—an in vitro study comparing a holmium device to a novel pulsed solid-state thulium laser. World Journal of Urology 39(9), 3651–3656 (2021)

[9] Plesset, M.S., Chapman, R.B.: Collapse of an initially spherical vapour cavity in the neighbourhood of a solid boundary. Journal of Fluid Mechanics 47(2), 283–290 (1971)

[10] Raboud, D.D., Westover, D.L.: Transient vibrations: Response of spring–mass system to a step function (2024). URL https://engcourses-uofa.ca/books/vibrations-and-sound/transient-vibrations/response-of-spring-mass-system-to-a-step-function/. Vibrations and Sound, Engineering at Alberta Courses, University of Alberta

[11] Supponen, O., Obreschkow, D., Tinguely, M., Kobel, P., Dorsaz, N., Farhat, M.: Scaling laws for jets of single cavitation bubbles. Journal of Fluid Mechanics 802, 263–293 (2016)

[12] Techet, A.: R2 lab 1: Added mass. In: Hydrodynamics —MIT Course No. 2.016. Massachusetts Institute of Technology, Cambridge MA (2005). URL https://ocw.mit.edu/courses/2-016-hydrodynamics-13-012-fall-2005/resources/lab1/. MIT OpenCourseWare

[13] Teichman, J.M.H., Qiu, J., Kang, W., Chan, K.F., Milner, T.E.: Laser Lithotripsy Physics, pp. 313–325. Springer London, London (2012)

[14] Tomita, Y., Robinson, P.B., Tong, R.P., Blake, J.R.: Growth and collapse of cavitation bubbles near a curved rigid boundary. Journal of Fluid Mechanics 466, 259–283 (2002)

[15] Xiang, G., Li, D., Chen, J., Mishra, A., Sankin, G., Zhao, X., Tang, Y., Wang, K., Yao, J., Zhong, P.: Dissimilar cavitation dynamics and damage patterns produced by parallel fiber alignment to the stone surface in holmium:yttrium aluminum garnet laser lithotripsy. Physics of Fluids 35(3), 033,303 (2023)

[16] Zhao, X., Ma, W., Chen, J., Xiang, G., Zhong, P., Wang, K.: Vapour bubbles produced by long-pulsed laser: a race between advection and phase transition. Journal of Fluid Mechanics 999, A103 (2024)
